# Investigation of the cytotoxicity of bioinspired coumarin analogues towards human breast cancer cells

**DOI:** 10.1007/s11030-020-10082-6

**Published:** 2020-04-23

**Authors:** Leonidas Gkionis, Eleni Kavetsou, Alexandros Kalospyros, Dimitris Manousakis, Miguel Garzon Sanz, Sam Butterworth, Anastasia Detsi, Annalisa Tirella

**Affiliations:** 1 Division of Pharmacy and Optometry, Faculty of Biology, Medicine and Health, Manchester Academic Health Science Centre, University of Manchester, Oxford Road, Manchester, M13 9PL UK; 2 Laboratory of Organic Chemistry, School of Chemical Engineering, National Technical University of Athens, Heroon Polytechniou 9, Zografou Campus, 15780 Athens, Greece; 3 NorthWest Centre for Advanced Drug Delivery (NoWCADD), Faculty of Biology, Medicine and Health, University of Manchester, Oxford Road, Manchester, M13 9PT UK

**Keywords:** Alkoxy-coumarins, Auraptene, Umbelliprenin, Breast cancer

## Abstract

**Abstract:**

Coumarins possess a wide array of therapeutic capabilities, but often with unclear mechanism of action. We tested a small library of 18 coumarin derivatives against human invasive breast ductal carcinoma cells with the capacity of each compound to inhibit cell proliferation scored, and the most potent coumarin analogues selected for further studies. Interestingly, the presence of two prenyloxy groups (5,7-diprenyloxy-4-methyl-coumarin, **4g**) or the presence of octyloxy substituent (coumarin **4d**) was found to increase the potency of compounds in breast cancer cells, but not against healthy human fibroblasts. The activity of potent compounds on breast cancer cells cultured more similarly to the conditions of the tumour microenvironment was also investigated, and increased toxicity was observed. Results suggest that tested coumarin derivatives could potentially reduce the growth of tumour mass. Moreover, their use as (combination) therapy in cancer treatment might have the potential of causing limited side effects.

**Graphic abstract:**

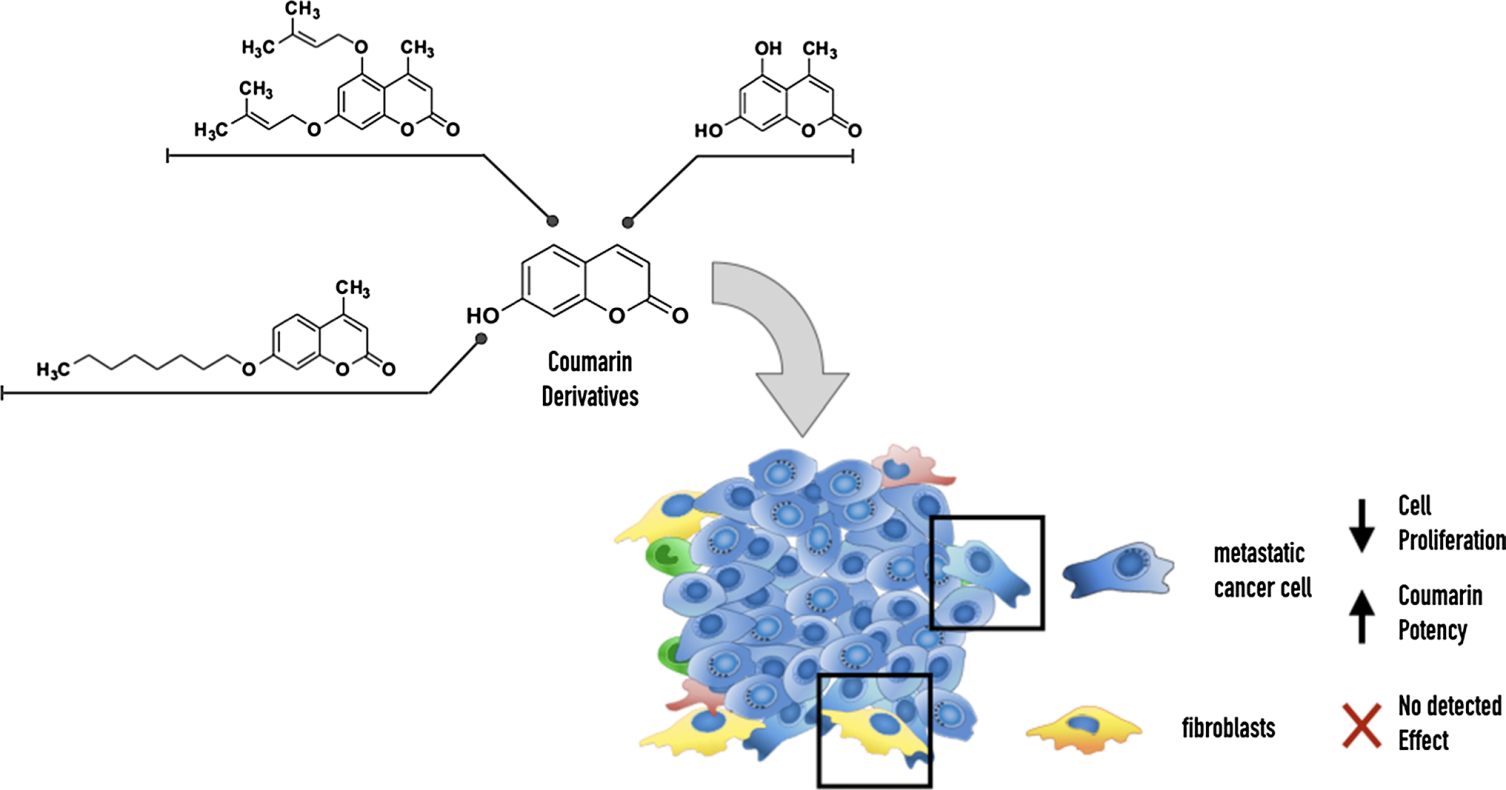

**Electronic supplementary material:**

The online version of this article (10.1007/s11030-020-10082-6) contains supplementary material, which is available to authorized users.

## Introduction

Breast carcinoma is considered the predominant and more common malignancy in women worldwide, with one in eight women potentially developing breast cancer during their lifetime [1] and predictions of 3.2 million newly diagnosed cases per year by 2050 [2]. Early detection and intervention are essential to increase patients’ survival rate, yet the treatment of advanced cancer remains an issue. While many biological and physicochemical factors have been identified in cancer development, there is an increasing interest in the role of inflammation and involvement of stromal component of the tumour microenvironment [3]. The challenge of treating breast cancer resides not only in the identification of active compounds capable of targeting the cancer, but mainly in identifying potent therapies with low side effects [4]. In this perspective, natural compounds like coumarins have gained significant interest in the recent years for their numerous pharmacological activities including chemopreventive and antiproliferative properties against various cancer types [5–8].

Coumarins are heterocyclic organic compounds that are widely distributed in nature [4, 9]. Coumarin derivatives have gained high scientific interest as promising drug candidates since they possess multiple pharmacological properties [10–13], such as antioxidant [14–16], antibacterial [17, 18], antimicrobial [18], antiviral [13], hepatoprotective [19] and anti-inflammatory effects [20–22]. Natural and synthetic coumarins have been also reported as effective chemopreventive and anticancer agents in vitro [23–26] and in vivo [27].

Chemical modification such as alkylation (the addition of unsaturated or saturated chain to the coumarin scaffold) has been shown to enhance the pharmacological profile of several coumarins, especially their anticancer activity [25]. In particular, the insertion of an unsaturated chain (prenyl, geranyl or farnesyl side chain) is known as prenylation and constitutes a metabolic pathway in nature (including plant kingdom and microorganism such as fungi and bacteria [28]). The process of prenylation is considered to further enhance the pharmacological activity of these metabolites mostly because it strengthens the lipophilicity of the molecules [29]. Recently, natural oxyprenylated coumarins (isopentenyloxy (C5), geranyloxy (C10) and farnesyloxy (C15) compounds and their biosynthetic derivatives) have been studied for their pharmacological properties [28], mainly as potential anticancer agents [30]. Auraptene (7-geranyloxy coumarin) and umbelliprenin are the most common plant-derived oxyprenylated coumarins, first isolated from citrus fruits and Ferula plant species, respectively, and present a wide range of bioactivities [25, 31–35]. The addition of an aliphatic chain to the coumarin scaffold is another modification shown to have anticancer effects as reported by Farley et al. [25], who reported that octyloxy-coumarins possess cytotoxicity against pancreatic cancer cells with concentrations in the order of tens of nΜ. As a continuation to our previous work concerning the biological evaluation of structurally modified coumarin analogues [14, 15], a series of bioinspired synthetic alkoxy coumarin derivatives (bearing saturated and unsaturated chains) were synthesized, structurally characterized and evaluated for their cytotoxicity against breast cancer cell lines (MCF-7 and MDA-MB-231) and fibroblasts. Interestingly we found that the more potent coumarin compounds have no effect on fibroblast (off-target control) and increase their potency on breast cancer cells cultured under nutrient-deprived conditions similar to the tumour microenvironment.

## Materials and methods

### Coumarins analogues

#### Synthesis

The chemicals used for synthesis and analysis were purchased from Sigma-Aldrich or Alfa Aesar (7-hydroxycoumarin, 98%) and used without further purification. NMR spectra were recorded on a Varian 300 MHz and 600 MHz spectrometer at the Institute of Chemical Biology of the National Hellenic Research Foundation. The HRMS spectra were obtained using a UHPLC-MSn Orbitrap Velos-Thermo mass spectrometer. Melting points were determined on a Gallenkamp MFB-595 melting point apparatus and are uncorrected.

##### General procedure for the synthesis of hydroxy or dihydroxy-4-substituted coumarin analogues

The desired compounds **3a** and **3b** were synthesized according to the method of Prousis et al. [36].

###### **7-Hydroxy-4-propyl-2H-chromen-2-one (3a)**

Beige solid; yield 80% (735.4 mg, 3.60 mmol); mp 130 °C (lit. m.p. 127–128 °C) [36]. ^1^H ΝMR (600 MHz, DMSO-*d*_*6*_): *δ*(ppm) 10.51 (s, 1H, 7-OH), 7.64 (d, *J* = 9.0 Hz, 1H, H-5), 6.79 (d, *J* = 8.4 Hz, 1H, H-6), 6.71 (s, 1H, H-8), 6.08 (s, 1H, H-3), 2.70 (t, *J* = 7.5 Hz, 2H, 4-C***H***_***2***_CH_2_CH_3_), 1.62 (m, 2H, 4-CH_2_C***H***_***2***_CH_3_), 0.96 (t, *J* = 7.2 Hz, 3H, 4-CH_2_CH_2_***CH***_***3***_).

###### **5,7-Dihydroxy-4-methyl-2H-chromen-2-one (3b)**

Beige solid; yield 93% (707.2 mg, 3.68 mmol); mp 288–289 °C (lit. m.p. 289–290 °C) [36]. ^1^H ΝMR (600 MHz, DMSO-*d*_*6*_): *δ*(ppm) 10.51 (s, 1H, 7-OH), 10.29 (s, 1H, 5-OH), 6.25 (s,1H, H-8), 6.16 (s, 1H, H-6), 5.84 (s, 1H, H-3), 2.48 (d, *J *=9.6 Hz, 3H, 4-CH_3_).

##### Synthesis of geranylgeranyl iodide

The following method was adapted from Alvarez-Manzaneda et al. [37]; briefly, 1170.0 mg (1.72 mmol, 1 eq.) of imidazole and 450.0 mg (1.72 mmol, 1 eq.) of triphenylphosphine were dissolved in 10 mL of anhydrous dichloromethane (DCM) in a round-bottom flask. 435.0 mg (1.72 mol, 1 eq.) of iodine was added slowly, and the mixture was stirred for 30 min. Then, the flask was covered with aluminium foil and placed in an ice bath, followed by slow addition of 0.57 mL (1.72 mol, 1 eq.) of geranylgeranyl. The mixture was stirred for approximately 2 h. After the reaction was complete (monitored by TLC in pure hexane), the mixture was filtered through a plug of silica, which was then washed with pure hexane. The solvent was evaporated in *vacuo*, resulting in a dark oily film. Υield 52% (51.9 mg).

##### General procedure for the synthesis of alkoxy-coumarins **4a**–**4m**

One eq. of the hydroxy- or dihydroxy-4-substituted coumarins, **3a–3c**, and 1 eq. of potassium carbonate (K_2_CO_3_) were dissolved in dry acetone. Then, 1.2 eq. of the appropriate alkoxy-bromide or geranylgeranyl iodide was added dropwise at room temperature, and the mixture was refluxed for 6 h. After the completion of the reaction, K_2_CO_3_ was filtrated, the precipitate was washed with acetone and the solvent was removed in vacuo. The desired products were purified via silica gel column chromatography in a solvent system of petroleum ether/ethyl acetate (9:1). Diprenyloxy coumarins were obtained in high purity after two steps of silica gel chromatography.

###### **7-Prenyloxy-4-methyl-2H-chromen-2-one (4a)**

White solid; yield 70% (61.1 mg, 0.25 mmol); mp 84 °C (lit. m.p. 84–86 °C) [38].^1^H ΝMR (300 MHz, CDCl_3_): *δ*(ppm) 7.48 (d, *J *=8.7 Hz, 1H, H-5), 6.86 (dd, *J *=8.7 Hz & *J *=2.4 Hz, 1H, H-6), 6.82 (d, *J *=2.4 Hz, 1H, H-8), 5.48 (t, *J *=6.9 Hz, 1H, H-2′), 4.59 (d, *J *=6.9 Hz, 2H, H-1′), 2.42 (s, 3H, 4-CH_3_), 1.83 (s, 3H, 3′-CH_3_), 1.79 (s, 3H, 4′-CH_3_).

###### **7-Geranyloxy-4-methyl-2H-chromen-2-one (4b)** [14]

Brown gummy solid; yield 68% (296.8 mg, 0.95 mmol). ^1^H ΝMR (300 MHz, DMSO-*d*_*6*_): *δ*(ppm) 7.64 (d, *J* = 8.4 Hz, 1H, Η-5), 6.94–6.91 (m, 2H, H-6 & H-8), 6.18 (s, 1H, H-3), 5.42 (t, *J* = 6.0 Hz, 1H, H-2′), 5.03 (br, 1H, H-6′), 4.65 (d, *J* = 6.6 Hz, 2H, Η-1′), 2.39 (s, 3H, 4-CH_3_), 2.08–2.06 (m, 4H, H-4′ & H-5′), 1.73 (s, 3H, 3′-CH_3_), 1.61 (s, 3H, 8′-CH_3_), 1.56 (s, 3H, 7′-CH_3_).

###### **7-Farnesyloxy-4-methyl-2H-chromen-2-one (4c)**

Yellow gummy solid; yield 60% (319.6 mg, 0.84 mmol). ^1^H ΝMR (300 MHz, CDCl_3_): *δ*(ppm) 7.48 (d, *J* = 8.7 Hz, 1H, H-5), 6.86 (dd, *J* = 8.7 Hz & *J *=1.8 Hz, 1H, H-6), 6.82 (d, *J *=1.8 Hz, 1H, H-8), 6.13 (s, 1H, H-3), 5.47 (t, *J* = 6.9 Hz, 1H, H-2′), 5.10- 5.07 (m, 1H, H-6′), 4.60 (d, *J* = 6.6 Hz, 1H, H-1′), 2.39 (s, 3H, 4-CH_3_), 2.12–1.95 (m, 8H, H-4′ & H-5′ & H-8′ & H-9′), 1.76 (s, 3H, 3′-CH_3_), 1.67 (s, 3H, 7′-CH_3_), 1.59 (s, 6H, 11′-CH_3_ & 12′-CH_3_). ^13^C NMR (75 MHz, DMSO-*d*_*6*_): *δ*(ppm) 162.0, 160.6, 155.1, 153.9, 141.5, 135.1, 131.1, 126.8, 124.5, 123.9, 119.5, 113.5, 113.1, 111.5, 101.8, 65.6, 39.7, 39.3, 26.6, 26.0, 25.9, 18.6, 17.9, 16.9, 16.3. HRMS (ESI) calcd for C_25_H_32_O_3_Na: *m*/*z*: 403.2244, found: 403.2245.

###### **4-Methyl-7-octyloxy-2H-chromen-2-one (4d)**

Pale yellow solid; yield 55% (180 mg, 0.62 mmol); mp 51 °C (lit. m.p. 48–50 °C) [24].^1^H ΝMR(600 MHz, DMSO): *δ*(ppm) 8.58 (d, *J *= 8.4 Hz, 1H, H-5), 6.78 (dd, *J *= 9 Hz & *J* = 2.4 Hz, 1H, H-6), 6.68 (d, *J *= 2.4 Hz, 1H, H-8), 6.15 (s, 1H, H-3), 4.06 (t, *J *= 6.6 Hz, 2H, H-1′), 2.39 (s, 3H, 4-CH_3_), 1.72 (m, 2H, H-2′), 1.40 (m, 2H, H-7′), 1.29 (m, 8H, H-3′ & H-5′ & H-4′ & H-6′), 0.86 (t, *J *= 6.9 Hz, 3H, 7′-CH_3_).

###### **7-Prenyloxy-4-propyl-2H-chromen-2-one (4e)**

Yellow solid; yield 60% (135.0 mg, 0.49 mmol); mp 89 °C. ^1^H ΝMR (600 MHz, CDCl_3_): *δ*(ppm) 7.51 (d, *J* = 8.4 Hz, 1H, H-5), 6.85 (dd, *J* = 9.0 Hz & *J* = 2.4 Hz, 1H, H-6), 6.82 (d, *J* = 2.4 Hz, 1H, H-8), 6.11 (s, 1H, H-3), 5.47 (t, *J* = 6.9 Hz, 1H, H-2′), 4.57 (d, *J* = 7.2 Hz, 2H, H-1′), 2.69 (t, *J* = 7.5 Hz, 2H, 4-***CH***_***2***_CH_2_CH_3_), 1.80 (s, 3H, 3′-CH_3_), 1.76 (s, 3H, 4′-CH_3_), 1.72–1.69 (m, 2H, 4-CH_2_***CH***_***2***_CH_3_), 1.04 (t, *J* = 7.5 Hz, 3H, 4-CH_2_CH_2_***CH***_***3***_). ^13^C NMR (150 MHz, CDCl_3_): *δ*(ppm) 161.9, 161.7, 156.5, 155.6, 139.3, 125.4, 118.8, 113.0, 112.9, 110.9, 101.8, 77.6, 77.2, 76.7, 65.5, 33.9, 25.9, 21.6, 18.4, 14.1. HRMS (ESI) calcd for C_17_H_21_O_3_ (M + H)^+^: *m*/*z*: 273.1485, found: 273.1485.

###### **7-Octyloxy-4-propyl-2H-chromen-2-one (4f)**

Beige solid; yield 60% (350.0 mg, 1.11 mmol); mp 47 °C. ^1^H ΝMR (600 MHz, CDCl_3_): *δ*(ppm) 7.51 (d, *J* = 9.0 Hz, 1H, H-5), 6.84 (dd, *J* = 8.4 Hz & *J* = 1.8 Hz, 1H, H-6), 6.81 (d, *J* = 2.4 Hz, 1H, H-8), 6.12 (s, 1H, H-3), 4.01 (t, *J* = 6.6 Hz, 2H, H-1′), 2.71 (t, *J* = 7.5 Hz, 2H, 4-C***H***_***2***_CH_2_CH_3_), 1.80 (m, 2H, H-2′), 1.73 (m, 2H, 4-CH_2_***CH***_***2***_CH_3_), 1.45 (m, 2H, H-7′), 1.31 (m, 8H, H-3′ & H-5′ & H-4′ & H-6′), 1.04 (t, *J* = 7.2 Hz, 3H, 4-CH_2_CH_2_***CH***_***3***_), 0.88 (t, *J* = 6.9 Hz, 3H, 7′-CH_3_). ^13^C NMR (150 MHz, CDCl_3_): *δ*(ppm) 162.2, 161.8, 156.6, 155.7, 125.4, 112.9, 110.8, 101.6, 68.8, 33.9, 31.9, 29.4, 29.3, 29.1, 26.1, 22.8, 21.7, 14.2, 14.1. HRMS (APCI) calcd for C_20_H_29_O_3_(M + H)^+^: *m*/*z*: 317.2116, found: 317.2105.

###### **5,7-Diprenyloxy-4-methyl-2H-chromen-2-one (4** **g)**

Green solid; yield 40% (272.6 mg, 0.83 mmol); mp 90 °C. ^1^H ΝMR (600 MHz, CDCl_3_): *δ*(ppm) 6.43 (d, *J *=2.4 Hz, 1H, H-6), 6.31 (d, *J *=1.8 Hz, 1H, H-8), 5.93 (d, *J *=0.6 Hz, 1H, H-3), 5.47 (pseudotriplet, 2H, H-2′ & H-2″), 4.53 (dd, *J *=7.2 Hz & *J *=9.6 Hz, 4H, H-1′ & H-1″), 2.53 (s, 3H, 4-CH_3_), 1.81 (s, 3H, 3′-CH_3_), 1.80 (s, 3H, 4′-CH_3_), 1.76 (s, 3H, 3″-CH_3_), 1.73 (s, 3H, 4″-CH_3_). ^13^C NMR (150 MHz, CDCl_3_): *δ*(ppm) 162.1, 161.4, 158.4, 157.1, 154.8, 139.4, 138.7, 118.8, 111.3, 105.1, 96.9, 94.1, 65.9, 65.4, 25.9, 25.8, 24.5, 18.4, 18.3. HRMS (ESI) calcd for C_20_H_24_O_4_ (M + H)^+^: *m*/*z*: 351.1567, found: 351.1561.

###### **5,7-Diprenyloxy-4-propyl-2H-chromen-2-one (4** **h)**

White solid; yield 42% (272.5 mg, 0.76 mmol); mp 71–72 °C. ^1^H ΝMR (600 MHz, CDCl_3_): *δ*(ppm) 6.44 (d, *J *=2.4 Hz, 1H, H-6), 6.32 (d, *J *=1.8 Hz, 1H, H-8), 5.94 (s, 1H, H-3), 5.50 (t, *J *=7.2 Hz, 1H, H-2″), 5.47 (t, *J *=6.0 Hz, 1H, H-2″), 4.54 (d, *J *=6.6 Hz, 2H, H-1′), 4.51 (d, *J *=6.6 Hz, 2H, H-1″), 2.84 (t, *J *=7.8 Hz, 2H, 4-***CH***_***2***_CH_2_CH_3_), 1.81 (s, 6H, 3′-CH_3_ & 4′-CH_3_), 1.76 (s, 3H, 3″-CH_3_), 1.74 (s, 3H, 4″-CH_3_), 1.61–1.57 (m, 2H, 4-CH_2_***CH***_***2***_CH_3_), 0.97 (t, *J *=7.8 Hz, 3H, 4-CH_2_CH_2_***CH***_***3***_). ^13^C ΝMR (150 MHz, CDCl_3_): *δ*(ppm) 161.9, 161.5, 158.6, 158.0, 157.4, 139.4, 139.3, 118.8, 118.6, 110.8, 104.4, 96.9, 94.3, 65.7, 65.4, 38.8, 25.9, 25.8, 23.4, 18.4, 18.3, 13.9. HRMS (ESI) calcd for C_22_H_28_O_4_ (M + H)^+^: *m*/*z*: 379.1880, found: 379.1869.

###### **5,7-Digeranyloxy-4-propyl-2H-chromen-2-one (4i)**

White solid; yield 41% (262.6 mg, 0.53 mmol); mp 79 °C. ^1^H ΝMR (600 MHz, CDCl_3_): *δ*(ppm) 6.45 (d, *J *=1.8 Hz, 1H, H-6), 6.33 (d, *J *=1.8 Hz, 1H, H-8), 5.95 (s, 1H, H-3), 5.51 (t, *J *=6.6 Hz, 1H, H-1′), 5.46 (t, *J *=6.6 Hz, 1H, H-1″), 5.09 (pseudotriplet, 2H, H-6′ & H-6″), 4.57 (d, *J *=6.6 Hz, 2H, H-2′), 4.54 (d, *J *=6.6 Hz, 2H, H-2″), 2.85 (t, *J *=7.8 Hz, 2H, 4-C*H*_*2*_CH_2_CH_3_), 2.13–2.09 (m, 8H, H-4′, H-5′ & H-4″, H-5″), 1.76 (s, 3H, 3′-CH_3_), 1.73 (s, 3H, 3″-CH_3_), 1.68 (s, 3H, 7′-CH_3_), 1.67 (s, 3H, 8′-CH_3_), 1.61 (s, 6H, 7″-CH_3_ & 8″-CH_3_), 0.98 (t, *J *=7.8 Hz, 3H, 4-CH_2_CH_2_***CH***_***3***_). ^13^C NMR (150 MHz, CDCl_3_) :*δ*(ppm) 162.0, 161.6, 158.6, 158.0, 157.4, 142.5, 142.4, 132.1, 132.0, 123.8, 123.7, 118.6, 118.4, 110.8, 104.4, 96.9, 94.3, 65.8, 65.4, 39.7, 39.6, 38.8, 31.0, 26.4, 26.3, 25.8, 25.7, 23.3, 17.9, 17.8, 16.9, 16.8, 14.1. HRMS (APCI) calcd for C_32_H_45_O_4_ (M + H)^+^: *m*/*z*: 493.3312, found: 493.3302.

###### **7-Prenyloxy-2H-chromen-2-one (4j)**

White solid; yield 65% (59.9 mg, 0.26 mmol); mp 77 °C (lit. m.p. 77–78 °C) [39]. ^1^H ΝMR (300 MHz, CDCl_3_): *δ*(ppm) 7.63 (d, *J *=9.6 Hz, 1H, H-4), 7.36 (d, *J *=8.4 Hz, 1H, H-5), 6.86–6.82 (m, 2H, H-6 & H-8), 6.25 (d, *J *=9.3 Hz,1H, H-6), 5.48 (t, *J *=8.1 Hz, 1H, H-2′), 4.59 (d, *J *=6.9 Hz, 2H, H-1′), 1.84 (s, 3H, 3′-CH_3_), 1.79 (s, 3H, 4′-CH_3_).

###### **7-Geranyloxy-2H-chromen-2-one (auraptene) (4k)**

White solid; yield 60% (268.5 mg, 0.90 mmol); mp 63 °C (lit. m.p. 62–63 °C) [40]. ^1^H ΝMR (300 MHz, CDCl_3_): *δ*(ppm) 7.63 (d, *J* = 9.3 Hz, 1H, H-4), 7.36 (d, *J* = 8.4 Hz, 1H, H-5), 6.86–6.82 (m, 2H, H-6 & H-8), 6.24 (d, *J* = 9.3 Hz, 1H, H-3), 5.47 (t, *J* = 6 Hz, 1H, H-2′), 5.09 (t, *J* = 5.7 Hz, 1H, H-6′), 4.62 (d, *J* = 6.6 Hz, 2H, Η-1′), 2.15–2.11 (m, 4H, H-4′ & H-5′), 1.79 (s, 3H, 3′-CH_3_), 1.69 (s, 3H, 8′-CH_3_), 1.63 (s, 3H, 7′-CH_3_).

###### **7-Farnesyloxy-coumarin (umbelliprenin) (4l)**

Yellowish solid; yield 80% (439.8 mg, 1.20 mmol); mp 61 °C (lit. m.p. 58–60 °C) [40]. ^1^H ΝMR (300 MHz, CDCl_3_): *δ*(ppm) 7.62 (d, *J* = 9.3 Hz, 1H, H-4), 7.35 (d, *J* = 8.1 Hz, 1H, H-5), 6.86–6.82 (m, 2H, H-6 & H-8), 6.24 (d, *J* = 9.6 Hz, 1H, H-3), 5.48 (t, *J* = 6.6 Hz, 1H, H-2′), 4.62 (d, *J* = 6.6 Hz, 1H, H-1′), 2.18 -2.01 (m, 8H, H-4′ & H-5′ & H-8′ & H-9′), 1.79 (s, 3H, 3′-CH3), 1.70 (s, 3H, 8′-CH3), 1.63 (s, 6H, 11′-CH3 & 12′-CH3).

###### **7-Geranylgeranyloxy-coumarin (4m)**

[41] Yellow solid; yield 64% (365.0 mg, 0.94 mmol); mp 72–73 °C. ^1^H ΝMR (400 MHz, CDCl_3_): *δ*(ppm) 7.63 (d, 1H), 7.36 (d, 1H), 6.86–6.82 (m, 2H), 6.24 (d, 1H), 5.47 (td, 1H), 5.10–5.07 (m, 3H), 4.60 (d, 2H), 2.15–2.03 (m, 8H), 1.99–1.95 (m, 4H), 1.76 (s, 3H), 1.67 (s, 3H), 1.60 (s, 3H), 1.59 (s, 3H), 1.58 (s, 3H).

###### **5,7-Diacetyloxy-4-methyl-2H-chromen-2-one (5)**

1 eq of the coumarin **3b** and 2 eq of acetic anhydride were added to the appropriate amount of pyridine, and the mixture was heated at 80 °C. After the completion of the reaction, pyridine was removed in vacuo and a solid product was obtained. The final pure coumarin was selected after recrystallization. Green solid; yield 72% (273.5 mg, 0.99 mmol); mp 143–144 °C (lit. m.p. 150–151 °C) [42]. ^1^H ΝMR (600 MHz, CDCl_3_): *δ*(ppm) 7.06 (s, 1H, H-8), 6.87 (s, 1H, H-6), 6.20 (s, 1H, H-3), 2.50 (s, 3H, 4-CH_3_), 2.37 (s, 3H, 7-CH_3_CO), 2.32 (s, 3H, 5-CH_3_CO).

### Cell culture

Dulbecco’s modified Eagle’s medium (DMEM, D6429), foetal bovine serum (FBS, F9665), trypsin (T3924), l-glutamine (G7513), antibiotics (penicillin − streptomycin, P0781) and (3-(4,5-dimethylthiazol-2-yl)-2,5-diphenyltetrazolium bromide) (MTT, M2128) were purchased from Sigma-Aldrich (Gillingham, UK). Dulbecco’s modified Eagle’s medium with no glucose (DMEM, A1443001) was purchased from Gibco Thermo Fisher Scientific, UK. Human breast adenocarcinoma cell lines MCF-7 (HTB-22™) and MDA-MB-231 (HTB-26™) were kindly donated from Manchester Cancer Research Labs (University of Manchester, UK). Human colon fibroblasts 18-Co (CRL-1459™) were purchased from ATCC.

#### General cell culture

Unless otherwise specified, all cell culture experiments were performed in a humidified 5% (v/v) CO_2_ air atmosphere at 37 °C in complete medium, and cell culture growth media were supplemented with 10% (v/v) foetal bovine serum and 2 mM l-glutamine. Human breast adenocarcinoma cell lines were cultured, maintained at densities lower than 1 × 10^6^ cells/cm^2^ and discarded upon reaching passage number 60. Stromal healthy cells (human colorectal fibroblasts, 18Co) were cultured using complete DMEM medium supplemented also with 1% (v/v) penicillin–streptomycin. Cells were maintained at densities less than 1 × 10^6^ cells/cm^2^ and discarded upon reaching passage number 12.

#### Nutrient-deprived conditions

Cells were culture using nutrient-deprived cell culture conditions (i.e. cell culture media with no glucose, l-glutamine, HEPES and sodium pyruvate) to mimic conditions similar to the tumour microenvironment. Note that these experiments were performed only using breast cancer cells. MCF-7 and MDA-MB-231 cells were seeded in 96-well plates (Corning Inc., NY, USA) at a density of 1 × 10^4^ and 6.7 × 10^3^ cells/cm^2^, respectively. Cells were incubated with coumarin derivatives at concentrations of 0.1, 1, 10, 50, 100 and 250 μM up to 48 h. Untreated cells (negative) and cells incubated with 0.5% (v/v) DMSO in complete media (positive) were used as controls.

### Coumarins cytotoxicity

The full library of coumarins was tested in both breast cancer cell lines, i.e. MCF-7 and MDA-MB-231. Fibroblast were used as “healthy” control. Stocks of compounds **3a**–**3c**, **4a**–**4m** and **5** were dissolved in pure DMSO and then diluted in complete media. (Note that DMSO concentration was kept lower than 0.5% (v/v).) Briefly, MCF-7 and MDA-MB-231 cells were seeded in 96-well plates (3799, Corning Inc., NY, USA) at a density of 1 × 10^4^ and 6.7 × 10^3^ cells/cm^2^, respectively, whereas 18Co fibroblasts were seeded at a density of 1 × 10^4^ cells/cm^2^. Cells were incubated with coumarin derivatives at concentrations 0.1, 1, 10, 50, 100 and 250 μM for 48 h. Untreated cells (negative) and cells incubated with 0.5% (v/v) DMSO in complete media (positive) were used as controls.

#### Cell metabolism assay

For each treatment, cell viability was measured via MTT assay after 48-h incubation as following described. Cell culture medium was replaced with 150 μL of fresh medium and 30 μL of MTT solution, and cells were incubated for 4 h (37 °C, 5% CO_2_). After the formazan crystal formation, cell culture medium was removed from each well and replaced with 200 μL of DMSO. The absorbance was measured at 540-nm wavelength using a plate reader (Synergy 2 Biotek plate reader, Gen5 software).

#### Toxicity (IC_50_) and identification of potent coumarins

IC_50_ values were calculated (nonlinear regression, normalized response–variable slope) with GraphPad Prism (version 7.04). Values were ranked and classified as: high toxicity, moderate toxicity, poor toxicity and no toxicity. Threshold were set as 60, 80 and > 100 μM, respectively. The selection was used in order to test only the most toxic coumarins under cell culture condition more relevant to the tumour microenvironment, i.e. deprived cell culture media (Sect. 3.3).

## Results and discussion

### Design and synthesis of coumarin analogues

Our previous results concerning the cytotoxic activity evaluation of natural oxyprenylated coumarins [14] in combination with the latest literature data led us to design, a new series of diprenyloxy as well as dialkyloxy coumarins. The new series were designed in order to evaluate the influence of the disubstitution as well as the length of the lipophilic chain in the cytotoxicity against breast cancer cell lines.

**Scheme 1:**
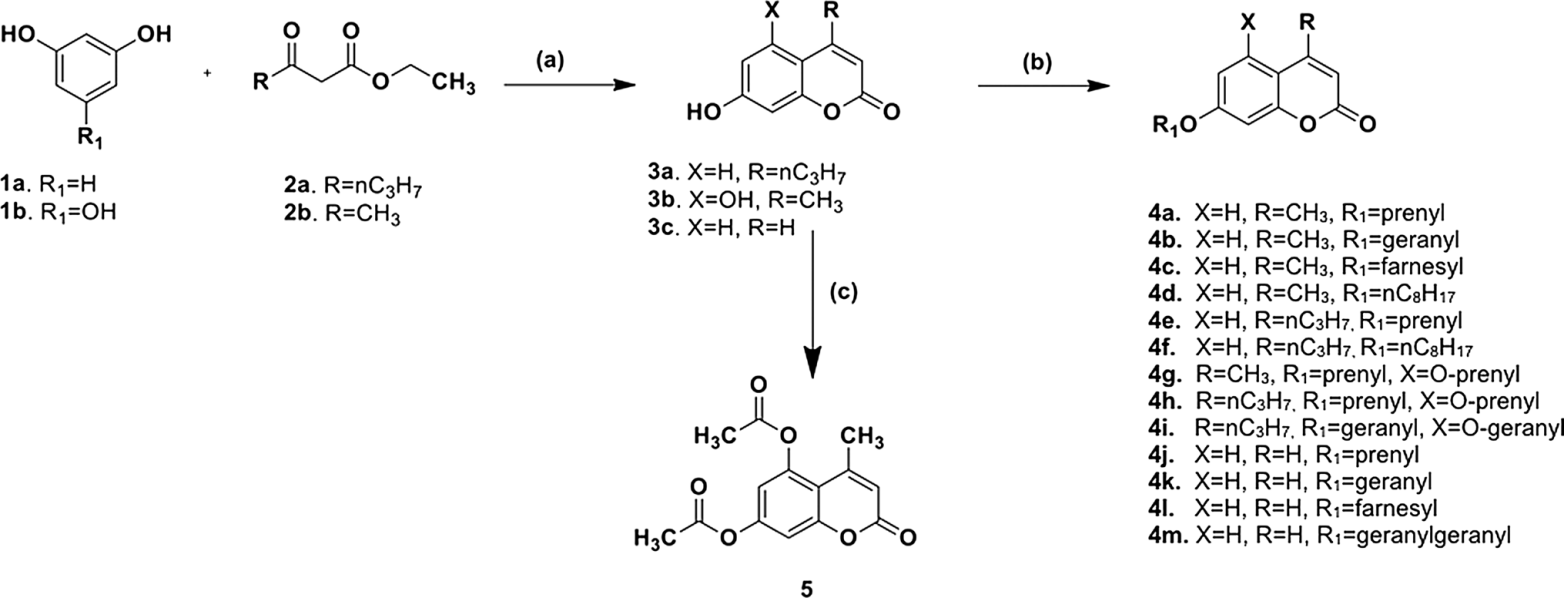
Synthesis of **3a**, **3b**, **4a**–**4m** and **5**; Reagents and conditions: **a** FeCl_3_/70 °C, 80–90%, **b** R_2_Br or geranylgeranyl iodide, K_2_CO_3_, acetone, 50–60%, **c** Ac_2_O, pyridine, 80 °C

In order to efficiently synthesize the desired coumarin analogues and the naturally occurring oxyprenylated coumarins, the appropriate hydroxy-4-substituted coumarins **3a** and **3b** were firstly synthesized via Pechmann reaction using iron (III) chloride (FeCl_3_) as the catalyst [36]. Compounds **3a** and **3b** as well as the commercially available 7-hydroxy-coumarin (umbelliferone, **3c**) were subsequently alkylated with the appropriate commercially available alkyl bromide using potassium carbonate (K_2_CO_3_) in acetone (Scheme 1). For the preparation of **4m**, the required geranylgeranyl iodide was prepared according to the method of Alvarez-Manzaneda et al. [37].

All the coumarin derivatives were purified using flash column chromatography and were structurally identified using ^1^H and ^13^C NMR spectroscopy and HRMS spectrometry.

### Cytotoxicity towards breast cancer cells

The systematic variations on the alkyl chain as well as the position of substitution at the coumarin scaffold were examined as potential factors which could affect anticancer activity. The first set of experiments identified the most potent candidates among the coumarin derivatives herein synthesized. Cytotoxicity was firstly evaluated on two breast cancer cell lines: MCF-7 and MDA-MB-231. MCF-7 cells were selected as they retain several characteristics of differentiated mammary epithelium proliferation, as well as expressing oestrogen receptor, whereas MDA-MB-231 was selected as expressing a more aggressive and metastatic cells that do not express high levels of the oestrogen, progesterone or HER2 receptors (i.e. triple negative).

Coumarins were classified as possessing high (IC_50_ < 80 µM), moderate (80 µM < IC_50_ < 100 µM) and poor toxicity (100 µM < IC_50_ < 250 µM); compounds with IC_50_ values > 250 µM were classified as non-toxic. Coumarin derivatives **4c**, **4d**, **4g, 4k** and **4l** were identified as the top-five most potent compounds tested in this study (Table 1) and were selected for further investigation.

**Table 1:**
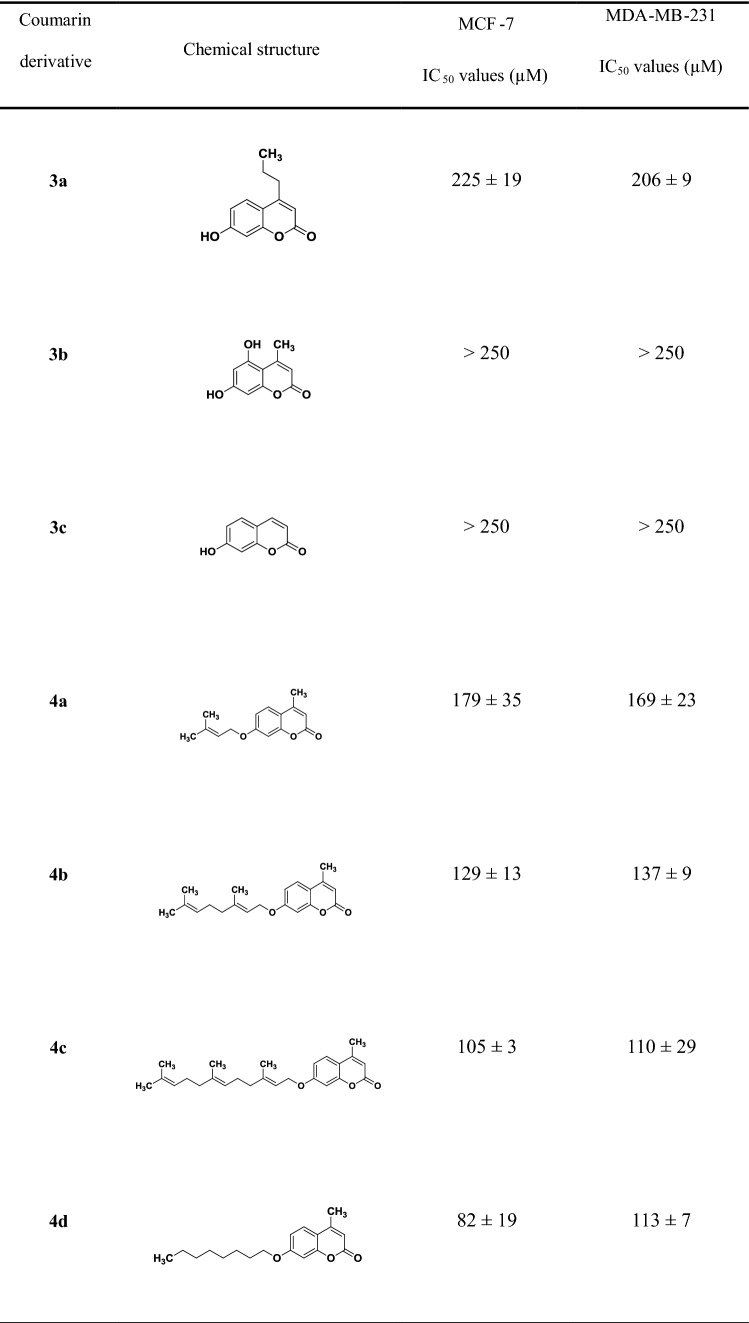

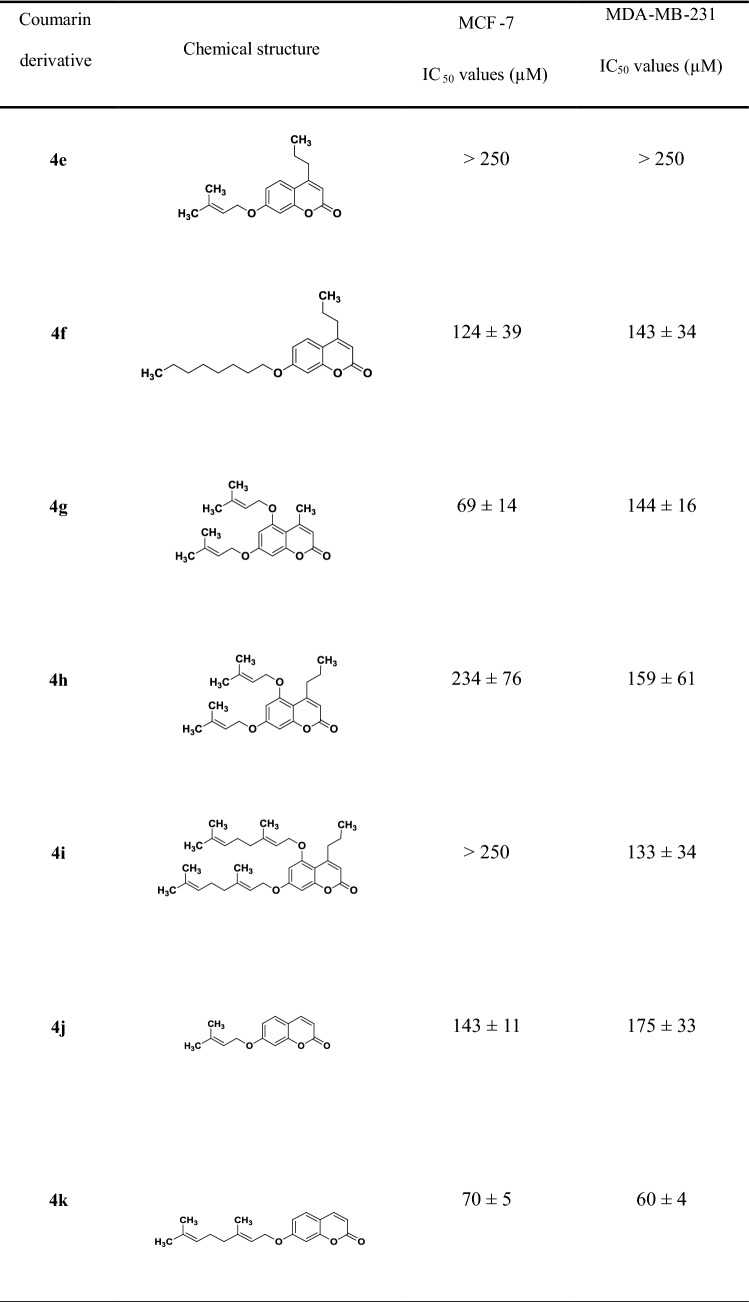

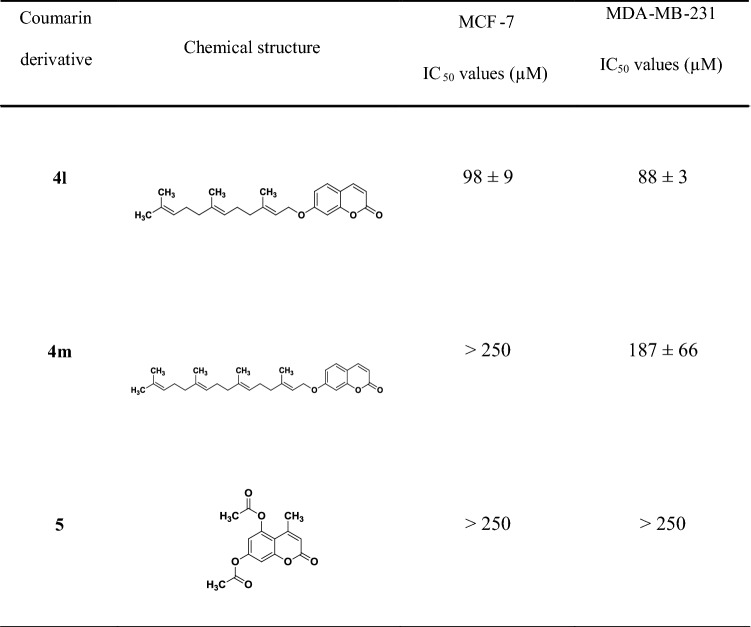
IC_50_ values (µM) of coumarin derivatives (18 compounds) on MCF-7 and MDA-MB-231 breast cancer cells obtained after 48-h incubation

Data are expressed as mean ± SD. of two independent experiments (*n* = 3 samples for each experiment). As control, MCF-7 and MDA-231 were incubated with [0.001–50] μg/mL of doxorubicin for 48 h, reporting IC_50_ values of (0.97 ± 0.60) μM and (0.48 ± 0.14) μM, respectively

One of the main drawbacks of cytotoxic compounds is the poor selectivity towards cancer cells, with undesired effects on healthy cells, e.g. fibroblasts and epithelia. In an effort to understand whether coumarins have any effect on ‘healthy’ cells, human fibroblasts (18-Co) were treated with the most potent coumarin derivatives. As control, fibroblasts were also treated with a non-toxic coumarin (i.e. umbelliferone, **3c**). Interestingly, cytotoxicity data evidenced no effect of the selected coumarins on fibroblasts (shown in Table 2) with the exception of coumarin **4k** that showed some toxicity towards ‘healthy’ cells. The tested compounds were not as toxic as typical chemotherapeutics with IC_50_s at the scale of few hundred nM such as doxorubicin [43–46] or gemcitabine [47–49], but they did appear to have no significant effects on ‘healthy’ fibroblasts as compared to the aforementioned chemo-agents. This is a very positive result in view of development of (nano)formulations and further translation of such compounds.

**Table 2:**
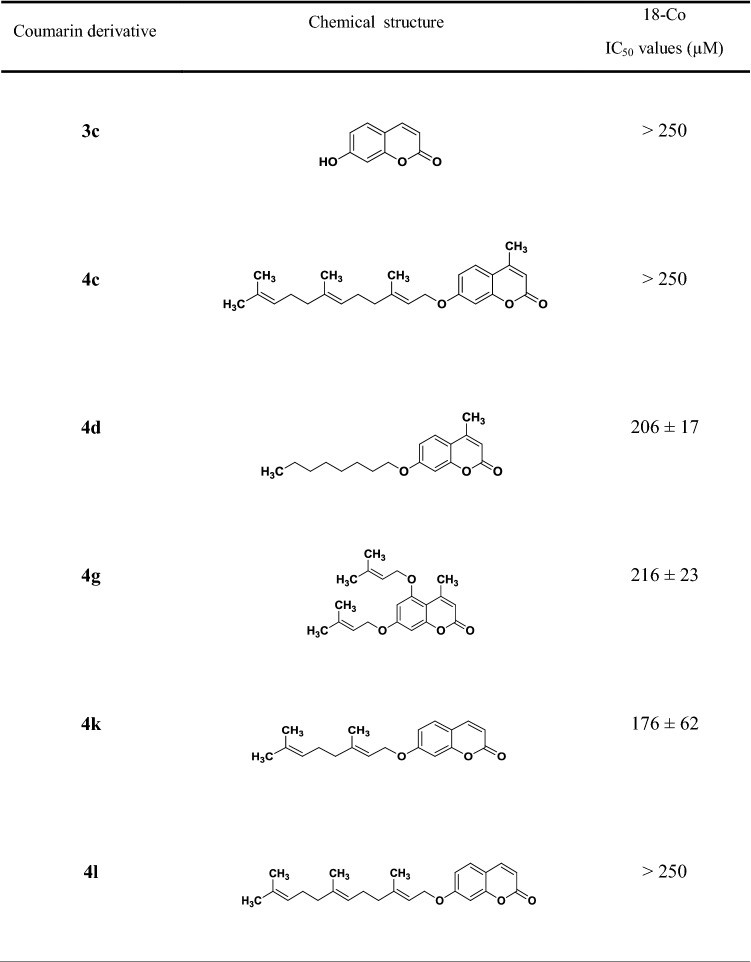
IC_50_ values (µM) of the most potent coumarin derivatives (five compounds: **4c**, **4d**, **4g**, **4k** and **4l**) on 18Co colon fibroblasts (healthy control)

Data are expressed as mean ± SD and are obtained from *n* = 2 independent experiments

**Fig. 1:**
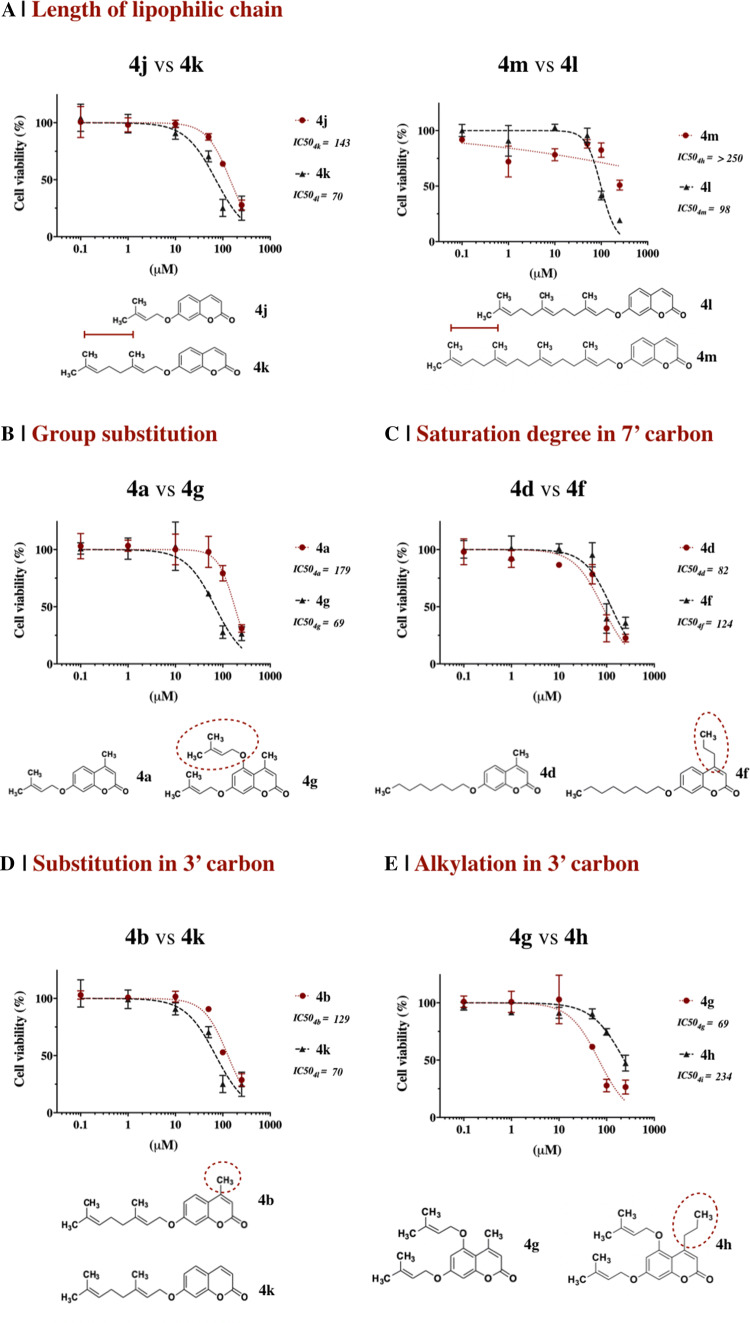
Effect of substituents on coumarins on MCF-7 cells: cell viability was measured after 48-h incubation with different concentrations of coumarins and IC_50_ values determined using nonlinear regression (GraphPad Prism, v7). Coumarins were compared on the basis of the following structural features: **a** length of lipophilic chain, coumarin **4k** versus **4j** (10 vs 5 carbons) and coumarin **4l** versus **4m** (15 vs 20 carbons); **b** position of the substituent on the coumarin scaffold, coumarin **4g** vs **4a**; **c** effect of the saturation degree (substitution at position C7), coumarin **4d** vs **4f**; **d** the presence of methyl group (substitution at position C4), coumarin **4b** versus **4k** and coumarin **4a** versus **4j** (data not shown), coumarin **4c** versus **4l** (data not shown); **e** the presence of propyl group (substitution at position C4), coumarins **4g** versus **4h** and coumarins **4d** versus **4f** (data not shown)

Auraptene (**4j**) was found to be the most potent compound among the library of coumarins tested in this study, confirming what has been already observed in other in vitro studies and in various in vivo animal models [50]. Its effect on cancer cells is still not clear and could be associated with induction of carcinogen-detoxifying enzymes, inhibition of free radical generation or metalloproteinase production [51]. The length of the prenyl chain seems to affect the activity of the compounds: auraptene (**4k**, 10 carbons) is more potent compared to its prenyloxy analogue (**4j**, 5 carbons) against the tested breast cancer cells. However, umbelliprenin (**4l**, 15 carbons) and coumarin **4m** (20 carbons) exhibited lower antitumour potency, with umbelliprenin being more toxic than coumarin **4m** in both cancer cell lines (Fig. 1).

The number of substituents on the aromatic ring could also play a role in the activity: 5,7-diprenyloxy-4-methyl-coumarin (**4g**) is approximately 2.5 times more cytotoxic compared to its monosubstituted analogue (compound **4a**) against MCF-7 cells. Increasing the chain length of the substituents, as in 7-geranylgeranyloxy-coumarin (**4n**), results in complete loss of activity against both cell lines (Fig. 1, Table 1).

In an effort to better investigate the role of unsaturation on cytotoxicity, the coumarin analogues **4d** and **4f,** which possess a saturated alkyloxy substituent, were synthesized. Only coumarin **4d** exhibited a moderate potency against MCF7 cells, whereas only a slight toxic effect was observed on MDA-MB-231 (Table 1); moreover, no toxicity was observed on fibroblasts (Table 2). These results suggest that this specific modification can participate in different biochemical pathways compared to unsaturated substituents; however, further research is necessary to confirm this and identify specific pathways.

Finally, we investigated the activity of derivatives as function of lipophilicity through the introduction of a methyl group at position 4 of the coumarin scaffold. The methylated coumarin derivatives **4b**, **4a** and **4c** exhibited a rather moderate cytotoxic effect compared to their corresponded non-methylated analogues **4k**, **4j** and **4l**. This observation suggests that substitution at position 4 might not directly link to increased toxicity. Furthermore, comparing the compounds with different substitutions at position4 (e.g. coumarins **4d** vs **4f**, coumarins **4g** vs **4h**) increased toxicity was observed in both cancer cell lines for compounds with methyl substitution (Fig. 1).

### Cytotoxicity in tumour relevant in vitro models

In accordance with the work of Jun et al. [52] and Devji et al. [41], we were motivated to assess the activity of some selected derivatives under nutrient-deprived conditions (NDCs). Cancer cells are programmed in a non-ordinary way to exhibit high glycolytic activity even under sufficient aerobic conditions [53]. In hypoxic tumour conditions, when oxygen depletion and low vascularization take place, cancer cells often find the way to proliferate rapidly by foregoing oxidative phosphorylation and instead ferment large amounts of glucose into lactate under aerobic glycolysis, known as the Warburg effect [54, 55]. Moreover, hypoxia tends to boost this phenomenon by up-regulating the HIF-1a factor that “switches on” glycolytic and glucose transporter gene expression [56]. Breast carcinoma cell lines behave in a glucose-dependent manner and derive the majority of energy needed from high-throughput glycolysis [56, 57]. Hyperglycaemic systemic conditions, i.e. diabetes, have been proved to further promote the migratory invasiveness of breast malignancies in patients [55].

On that basis, we exposed the breast cancer cells to nutrient-deprived conditions where culture media were supplemented only with 2.5% v/v FBS, but not additional glucose, l-glutamine, sodium pyruvate. The coumarins tested were auraptene (**4k**), umbelliprenin (**4l**) and analogues **4d**, **4c** and **4g**. As shown in Table 3, the tested compounds showed selective preferential cytotoxicity under nutrient-deprived conditions with umbelliprenin (**4l**) to be the most potent candidate as its pharmacological activity was remarkably enhanced by 15 times (IC_50_ = 9.0 and 7.0 for MCF7 and MDA-MB231 cells, respectively). In similar studies, Zhang et al. and Jun et al. reported the high preferential cytotoxicity of umbelliprenin (**4l**) and its C6 analogue under NDC against pancreatic cancer cells [52, 58]. It should be therefore noted that these derivatives could represent a potential new tool for treating aggressively metastatic hypoxic tumours. The exact mechanism of action, though, should be further investigated.

**Table 3:**
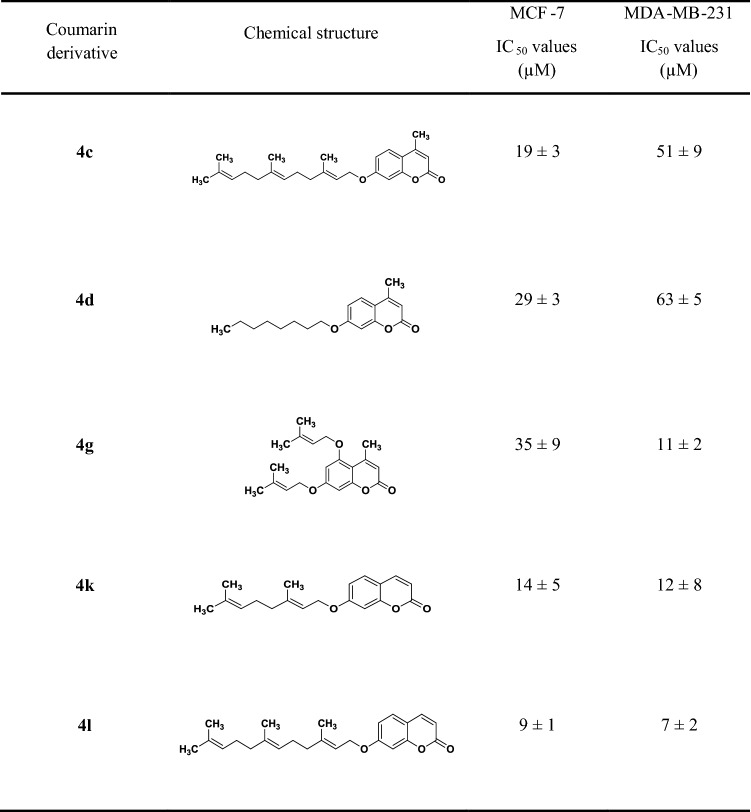
IC_50_ values (μM) of the tested coumarin derivatives **4c,4d, 4g, 4k** and **4l**. Values were obtained for both MCF-7 and MDA-MB-231 breast cancer cells cultured under nutrient-deprived conditions and incubated with coumarin derivatives up to 48 h

Data are expressed as average ± SD of *n* = 3 independent experiments

## Conclusions

A series of novel alkoxy-coumarin derivatives were synthesized and tested for their cytotoxicity against the MCF7 and MDA-MB-231 breast cancer cells. The results of this study indicate that alkylation modification induces noticeable differentiation in pharmacological activity of coumarins. Auraptene (**4k**) was found to possess the most potent cytotoxic activity among the tested derivatives followed by compounds **4c, 4d, 4g** and **4l**. The tested compounds seemed not to affect the cell viability of the healthy 18Co fibroblasts but for the highest dose only. The amplification of the cytotoxic effect of the above pharmacophores under nutrient-deprived conditions, with umbelliprenin (**4l**) being the lead compound, indicates that these compounds could lead to potential new therapeutics for highly metastatic hypoxic tumours once their mechanisms are fully understood.

## Electronic supplementary material

Below is the link to the electronic supplementary material.

Supplementary material 1 (DOCX 3770 kb)

## Data Availability

The raw data supporting the conclusions of this manuscript will be made available by the authors, without undue reservation, to any qualified researcher.
