# Supplementary material for: Investigation of the cytotoxicity of bioinspired coumarin analogues towards human breast cancer cells
Source: Mol Divers. 2020 Apr 23;25(1):307–21. doi: 10.1007/s11030-020-10082-6 (PMC7870773; doi:10.1007/s11030-020-10082-6)

**Investigation of the cytotoxicity of bioinspired coumarin analogues towards human breast cancer cells**

Leonidas Gkionis^1,§^, Eleni Kavetsou^2,§^, Alexandros Kalospyros^2^, Dimitris Manousakis^2^, Miguel Garzon sanz^1^, Sam Butterworth^1,3^, Anastasia Detsi^2,*^ and Annalisa Tirella^1,3,*^

^1^Division of Pharmacy and Optometry, Faculty of Biology, Medicine and Health, University of Manchester, Manchester Academic Health Science Centre, Oxford Road, Manchester M13 9PL, United Kingdom

^2^Laboratory of Organic Chemistry, School of Chemical Engineering, National Technical University of Athens, Heroon Polytechniou 9, Zografou Campus, GR 15780, Athens, Greece

^3^NorthWest Centre for Advanced Drug Delivery (NoWCADD), Faculty of Biology, Medicine and Health, University of Manchester, Oxford Road, Manchester, M13 9PT, United Kingdom

^§^Co-first authors

^*^Corresponding authors: annalisa.tirella@manchester.ac.uk, adetsi@chemeng.ntua.gr

**Supporting information**

**^1^H-NMR and ^13^C-NMR spectra for Compounds 3a-5**

**7-hydroxy-4-propyl-2H-chromen-2-one (3a)**

**^1^H NMR**


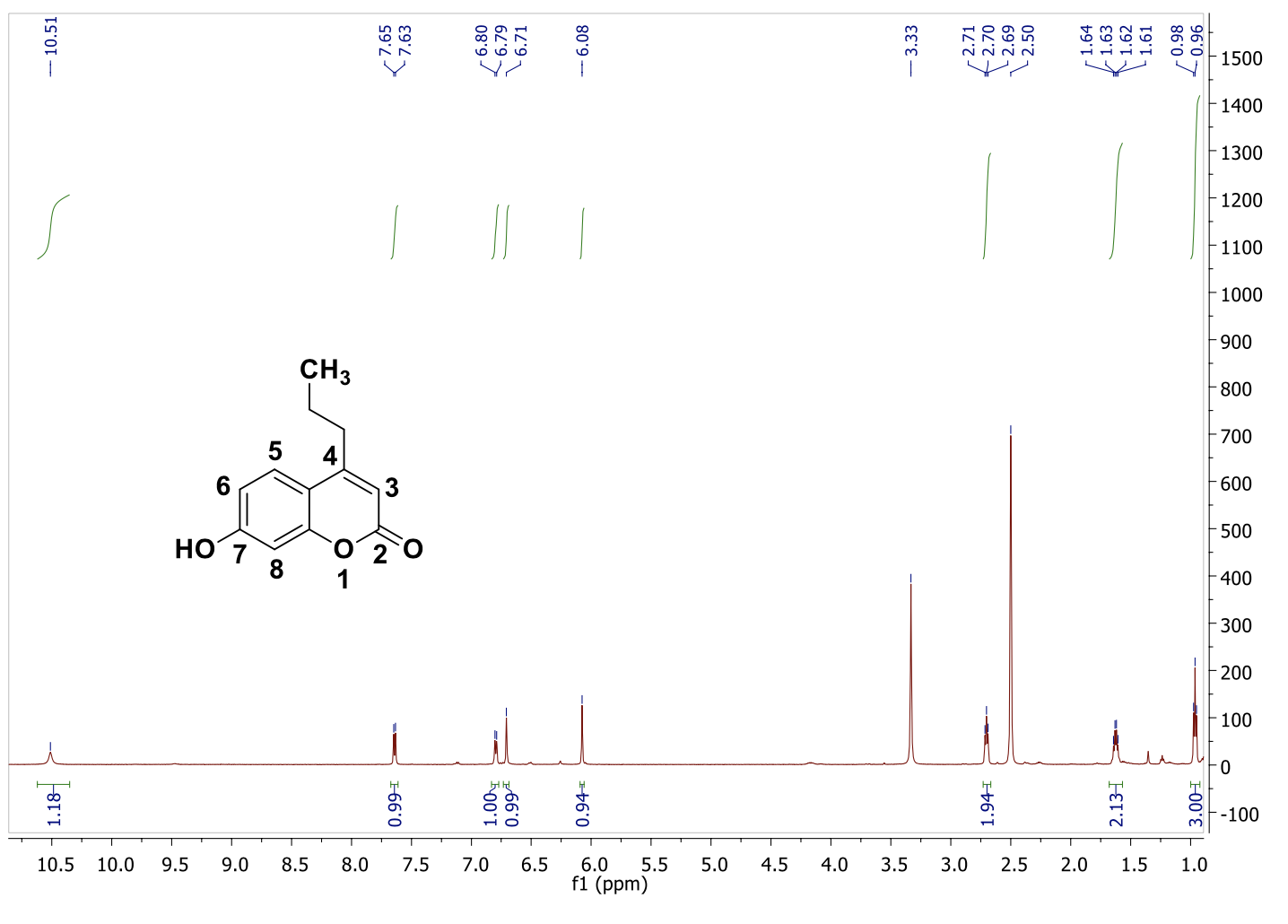


**5,7-dihydroxy-4-methyl-2H-chromen-2-one (3b)**

**^1^H NMR**


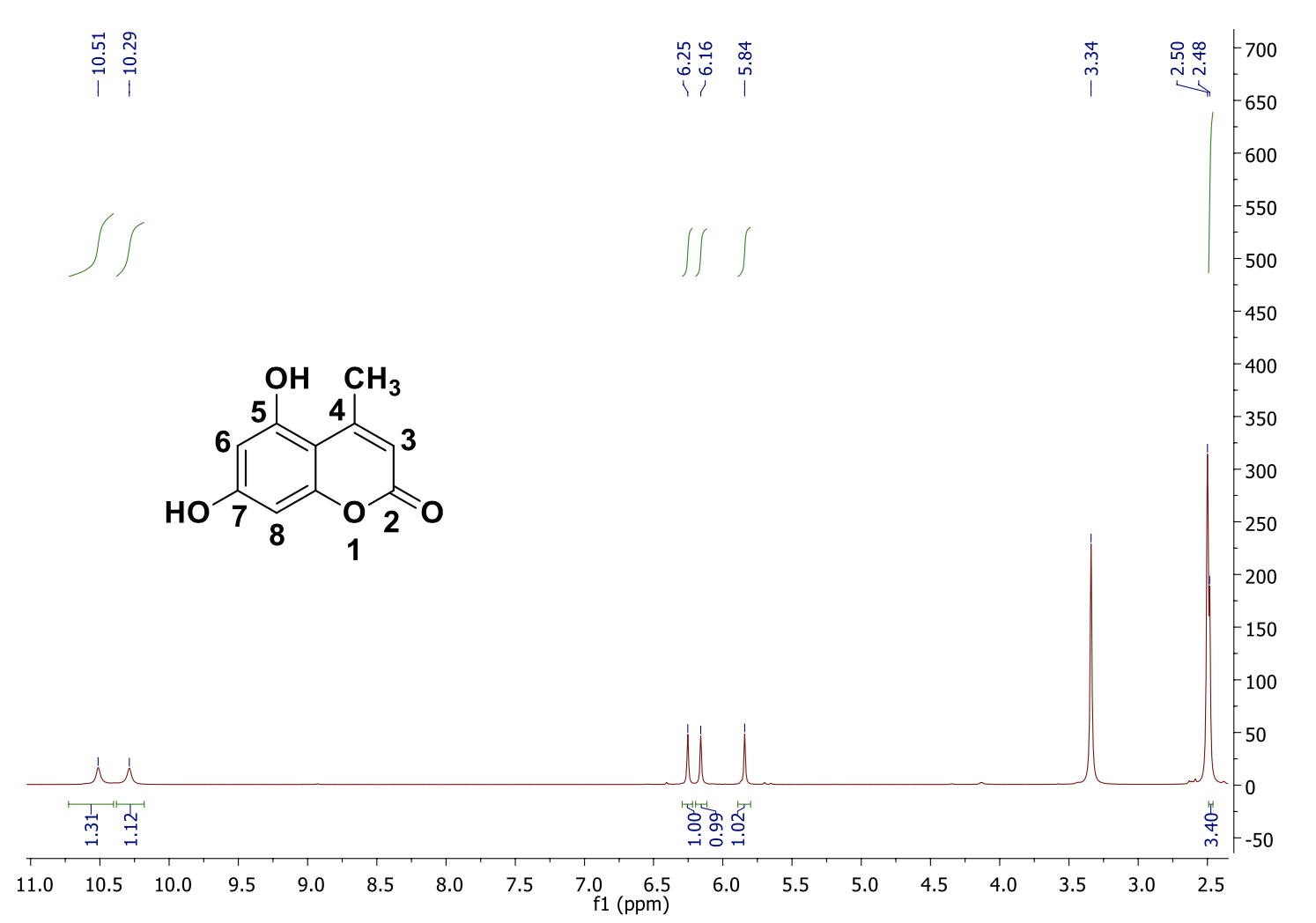


**7-prenyloxy-4-methyl-2H-chromen-2-one (4a)**

**^1^H NMR**


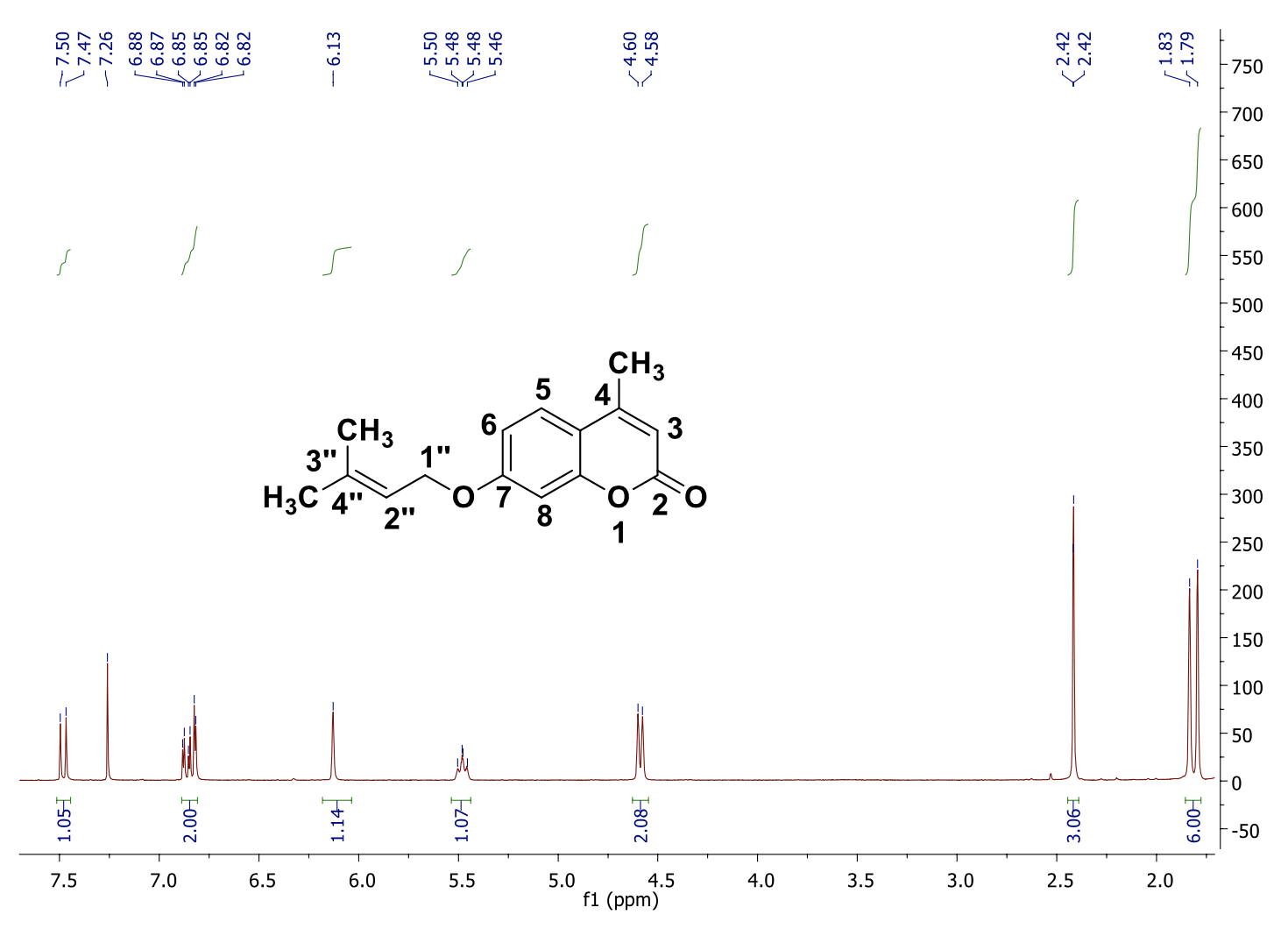


**7-geranyloxy-4-methyl-2H-chromen-2-one (4b)**

**^1^H NMR**


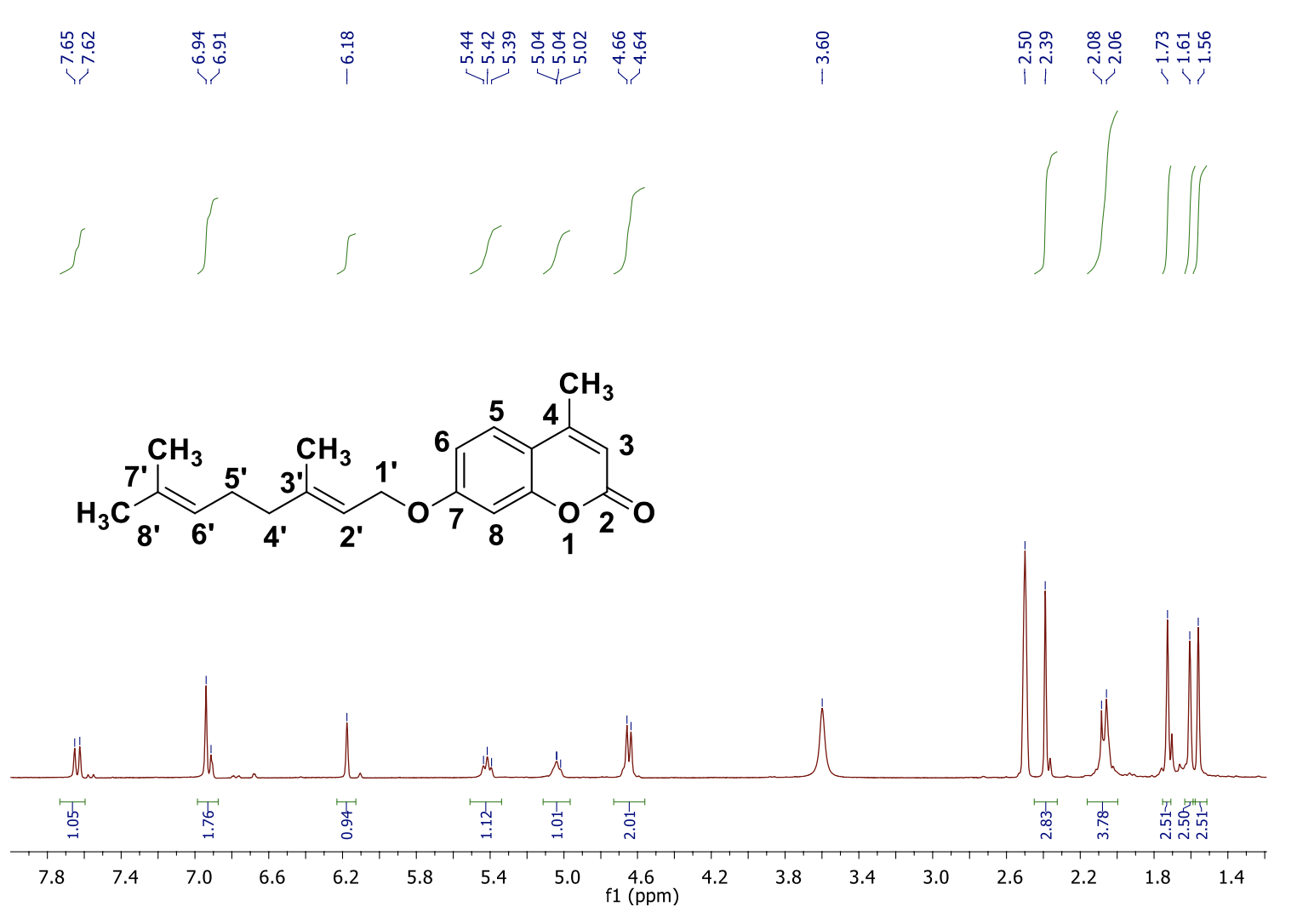


**7-farnesyloxy-4-methyl-2H-chromen-2-one (4c)**

**^1^H NMR**


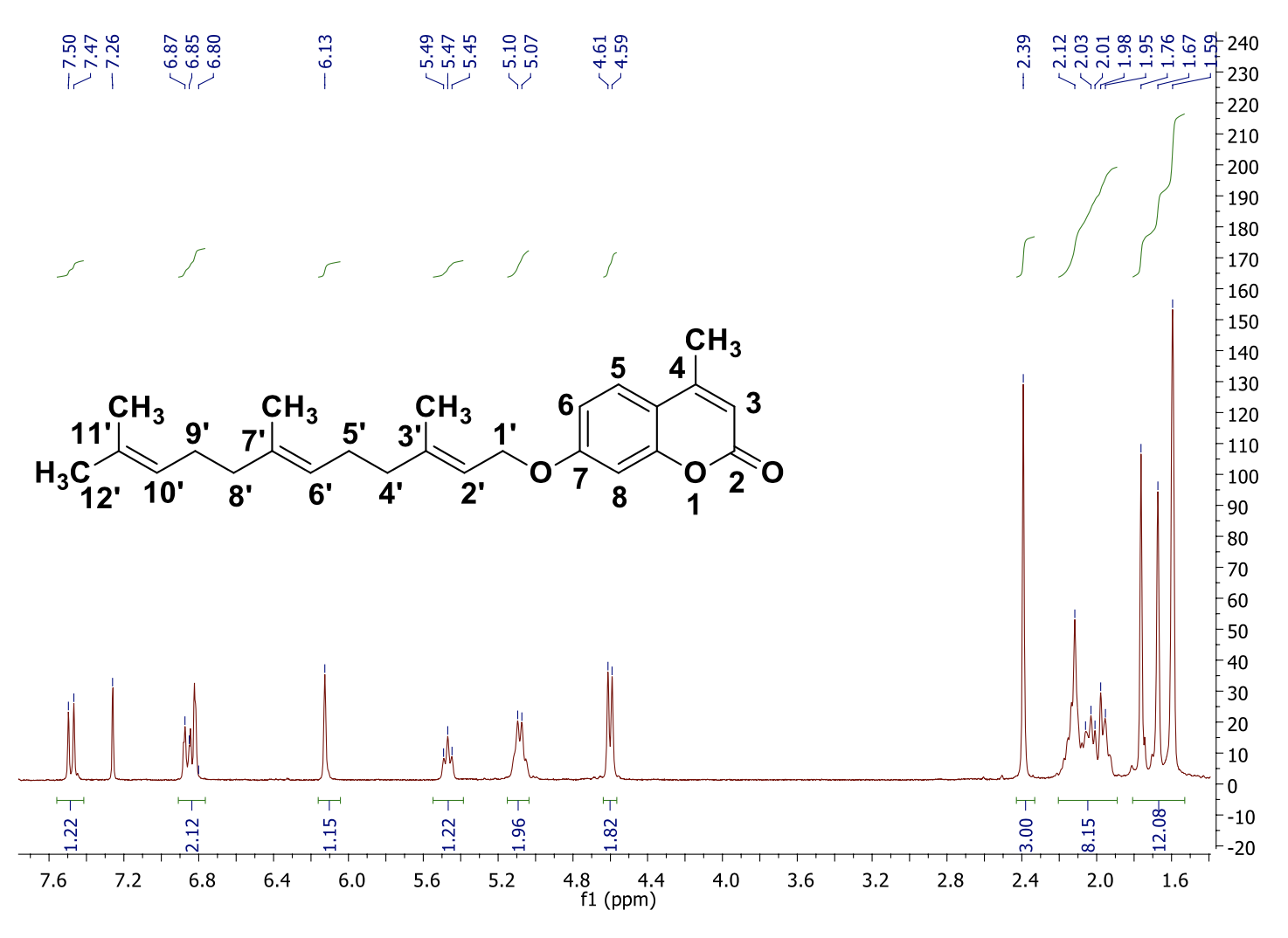


**^13^C NMR**


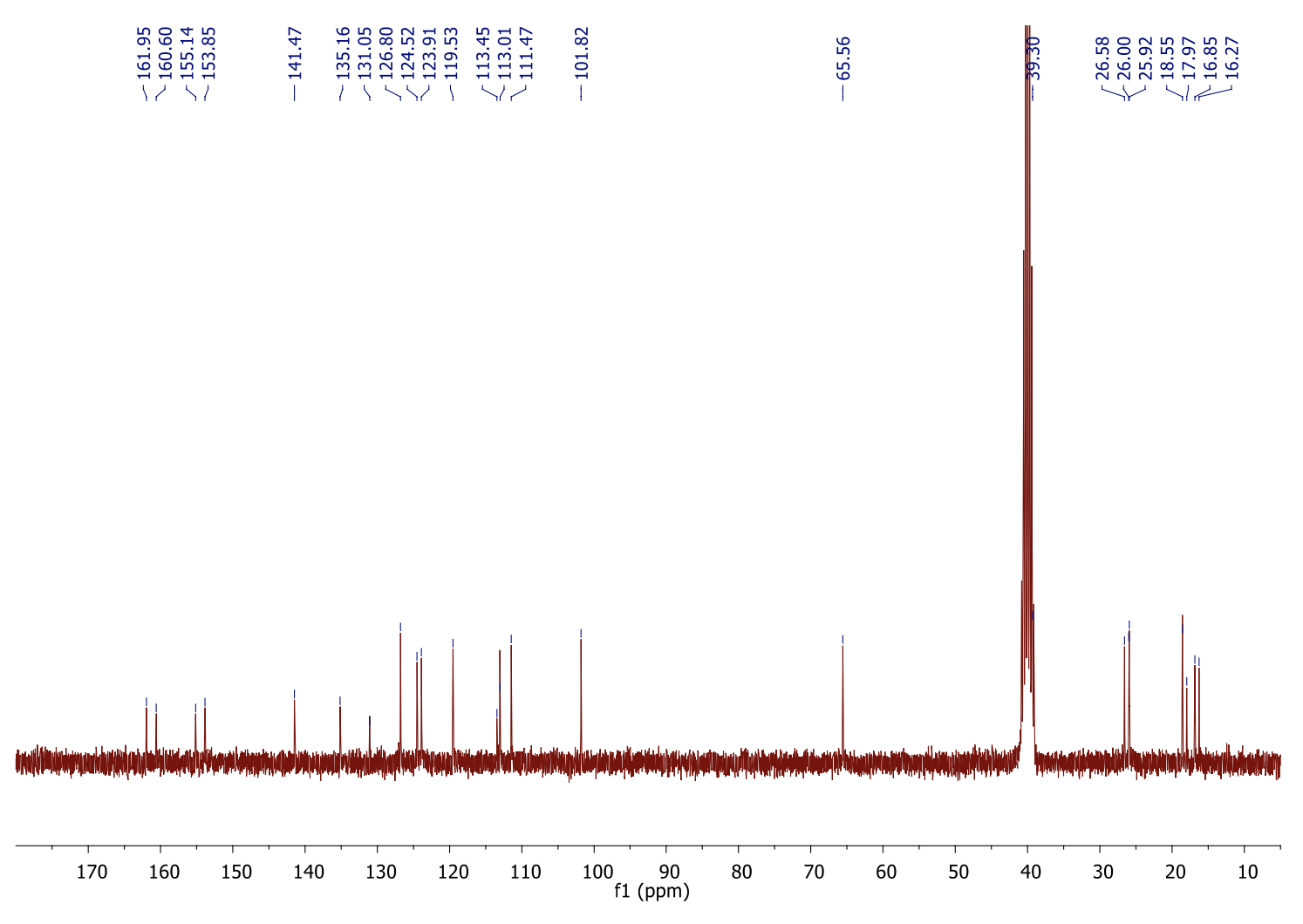


**7-prenyloxy-4-propyl-2H-chromen-2-one (4e)**

**^1^H NMR**


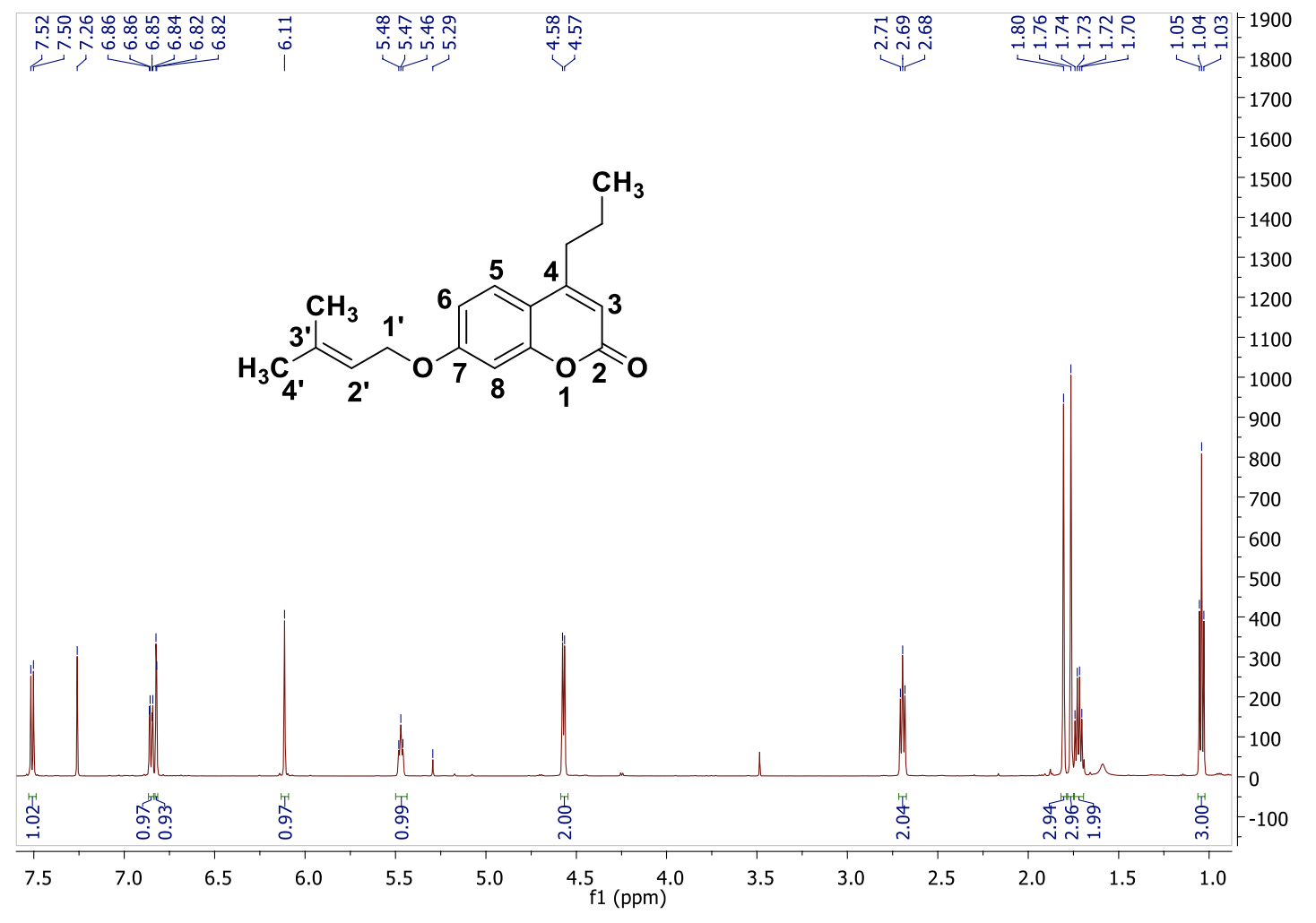


**^13^C NMR**


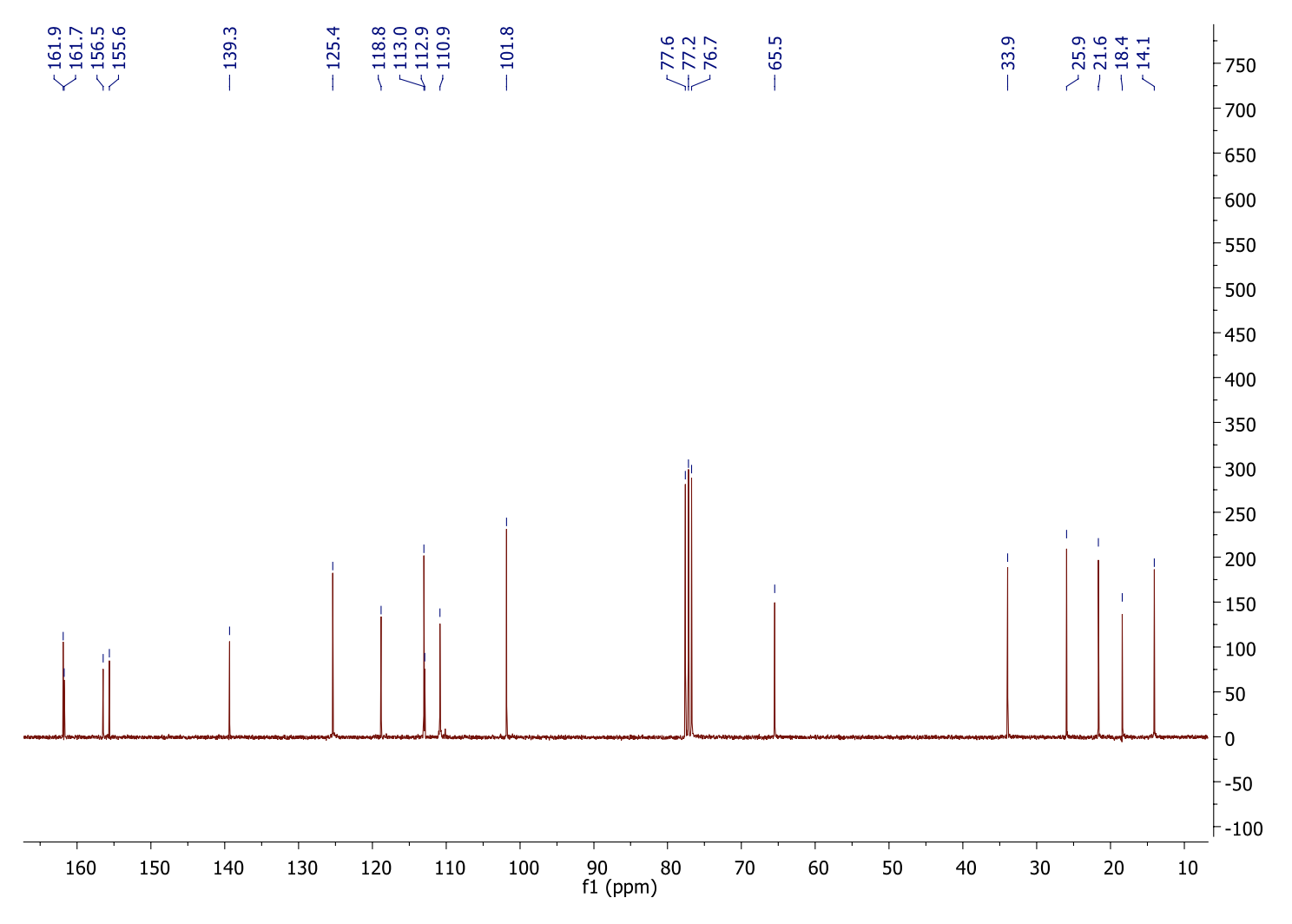


**7-octyloxy-4-propyl-2H-chromen-2-one (4f)**

**^1^H NMR**


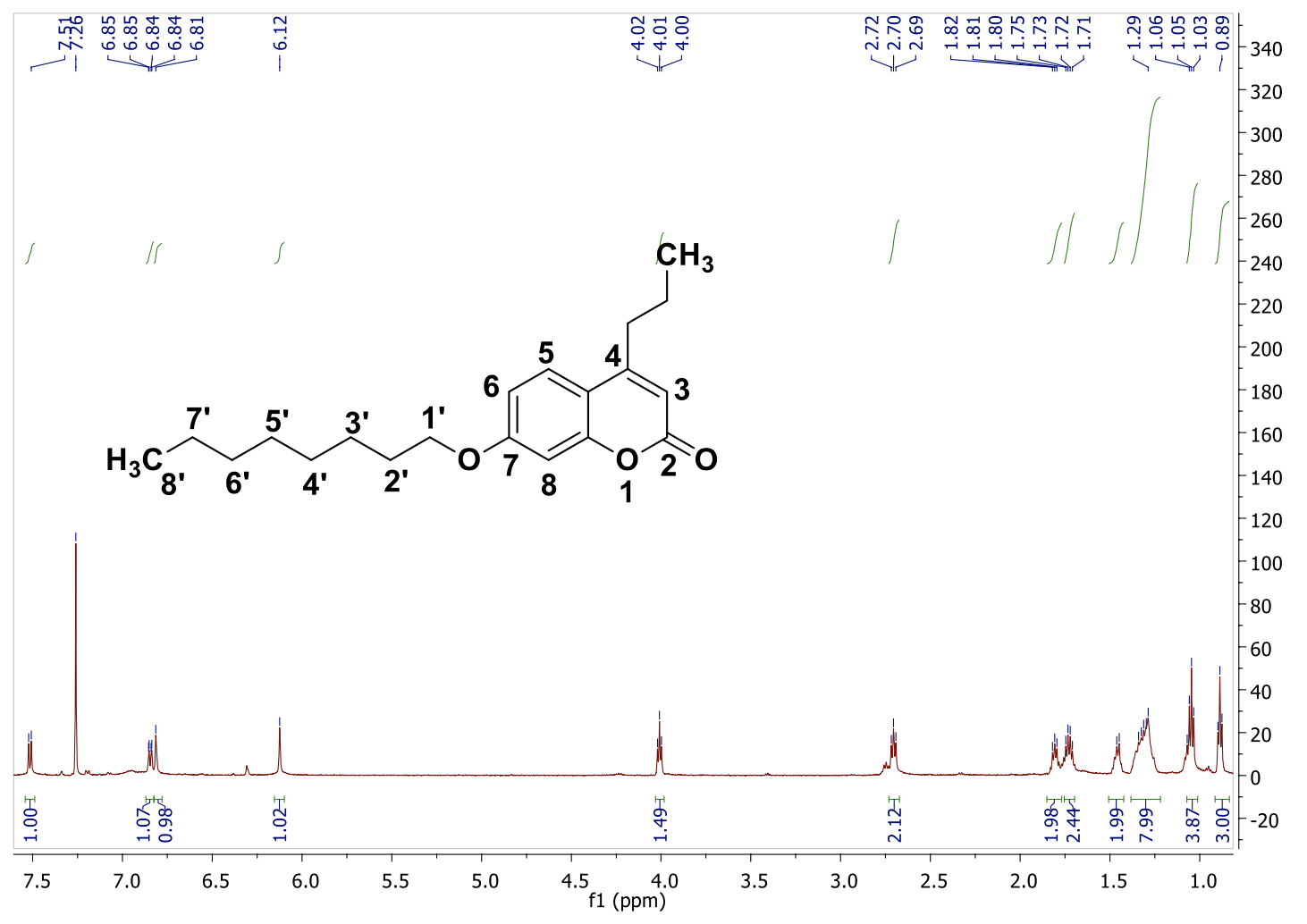


**^13^C NMR**


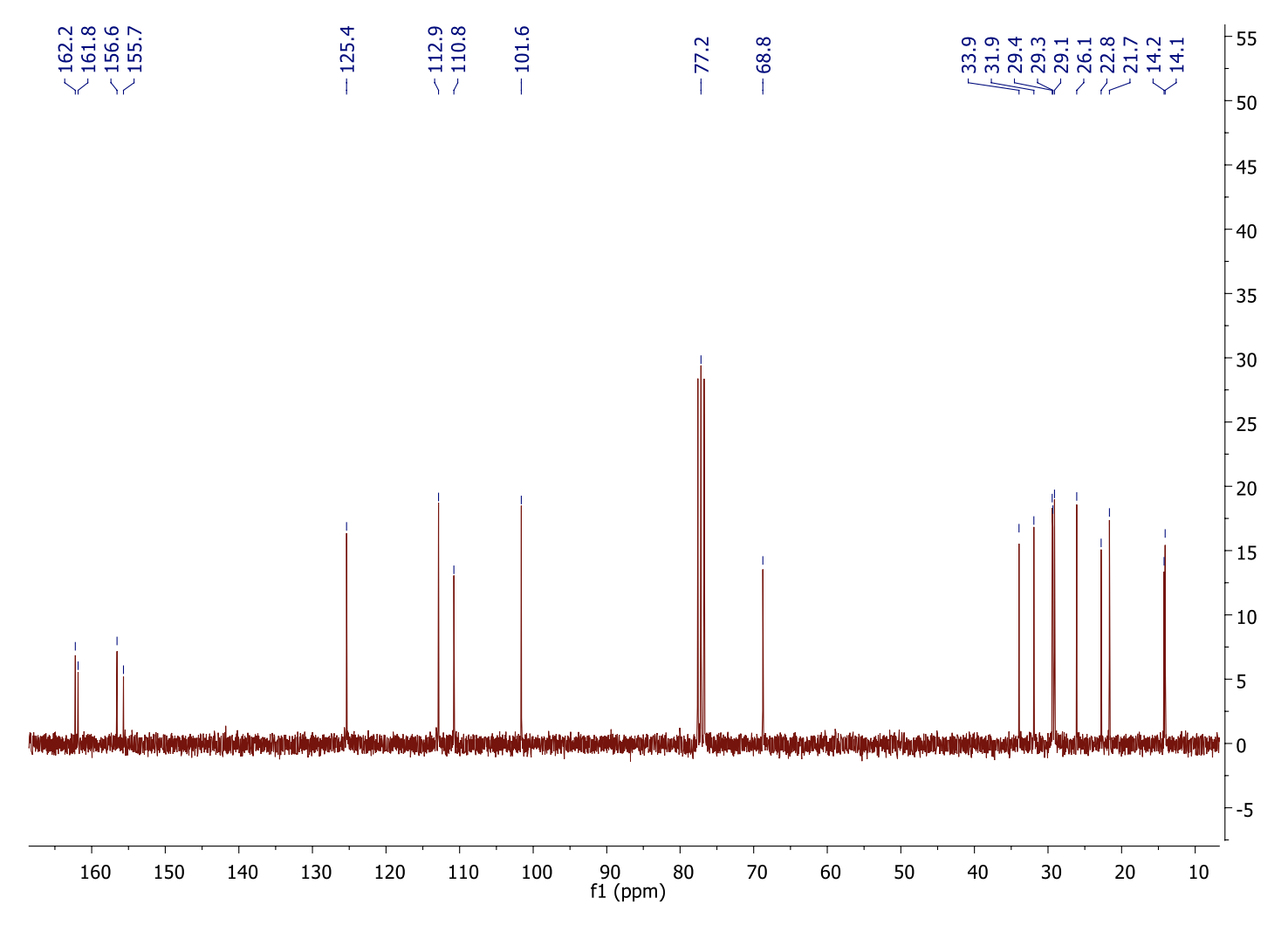


**5,7-diprenyloxy-4-methyl-2H-chromen-2-one (4g)**

**^1^H NMR**


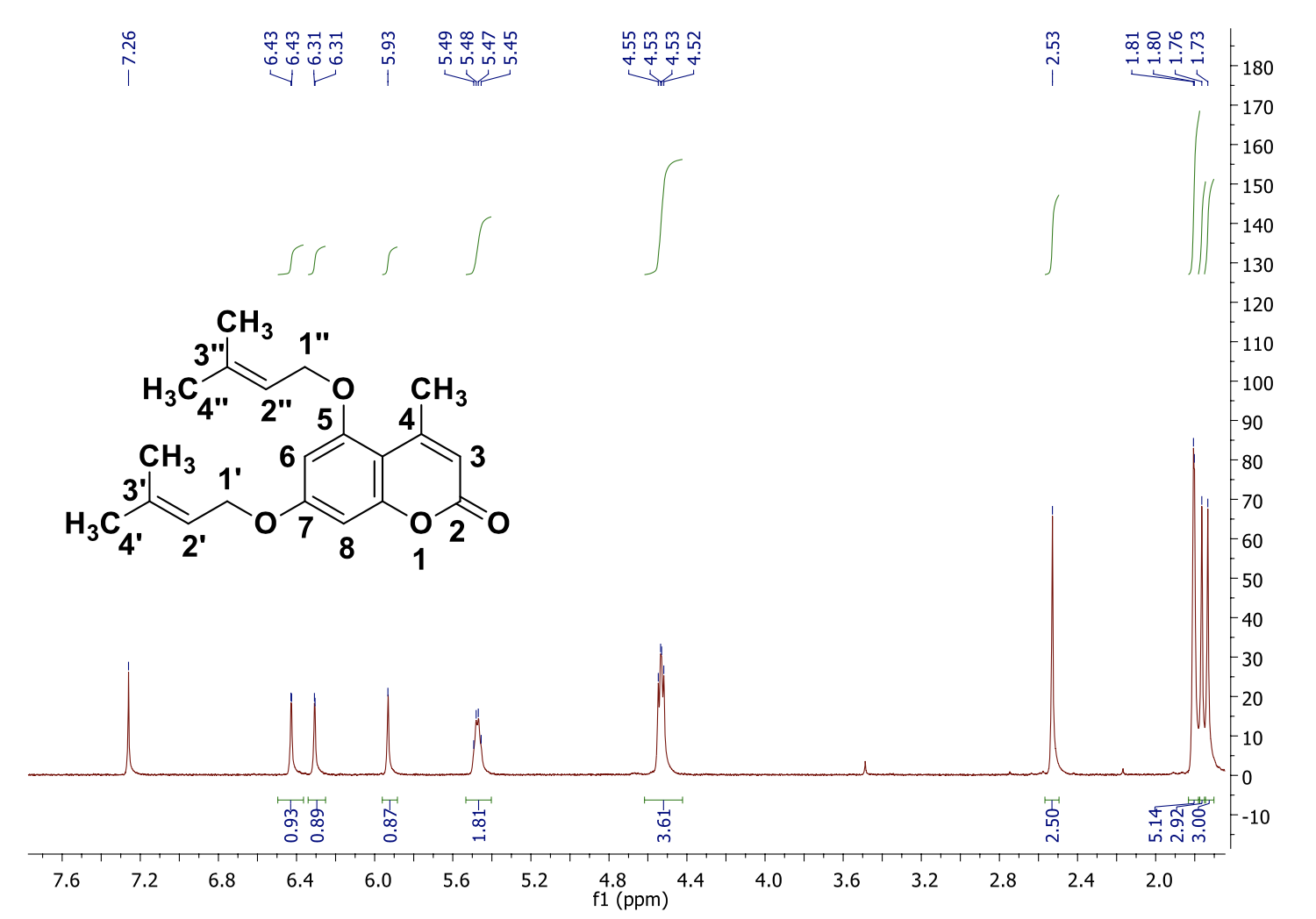


**^13^C NMR**


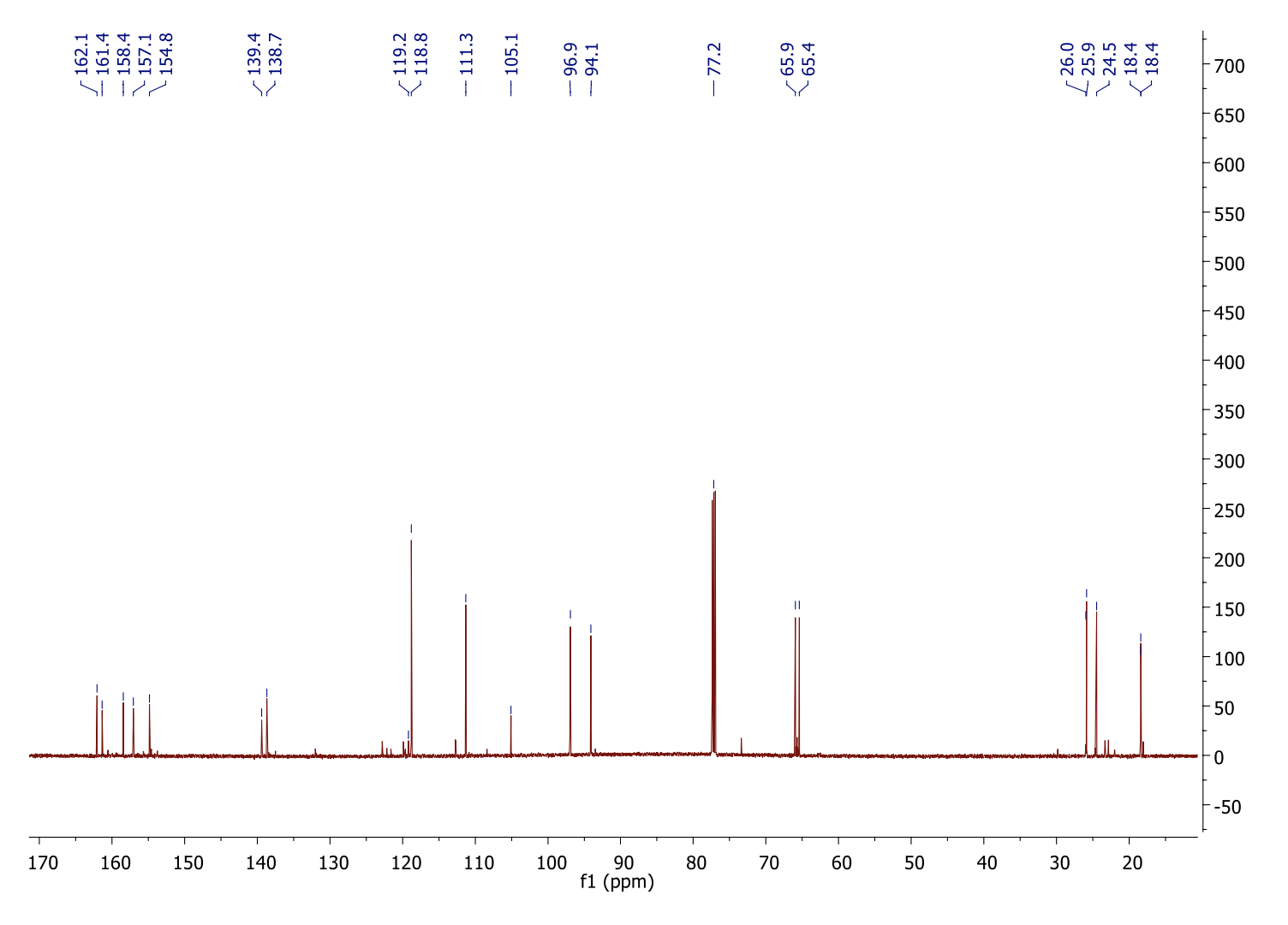


**5,7-diprenyloxy-4-propyl-2H-chromen-2-one (4h)**

**^1^H NMR**


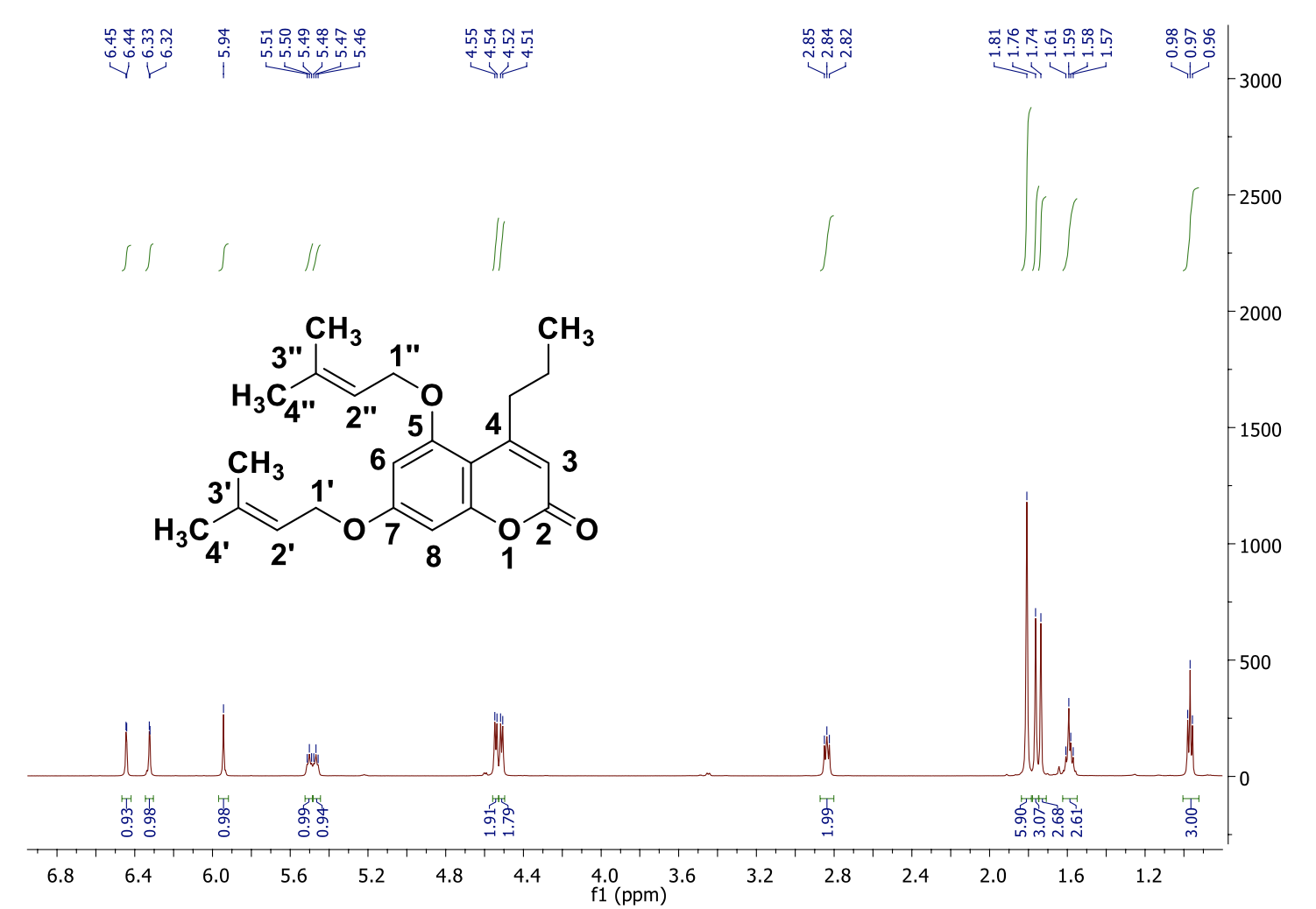


**^13^C NMR**


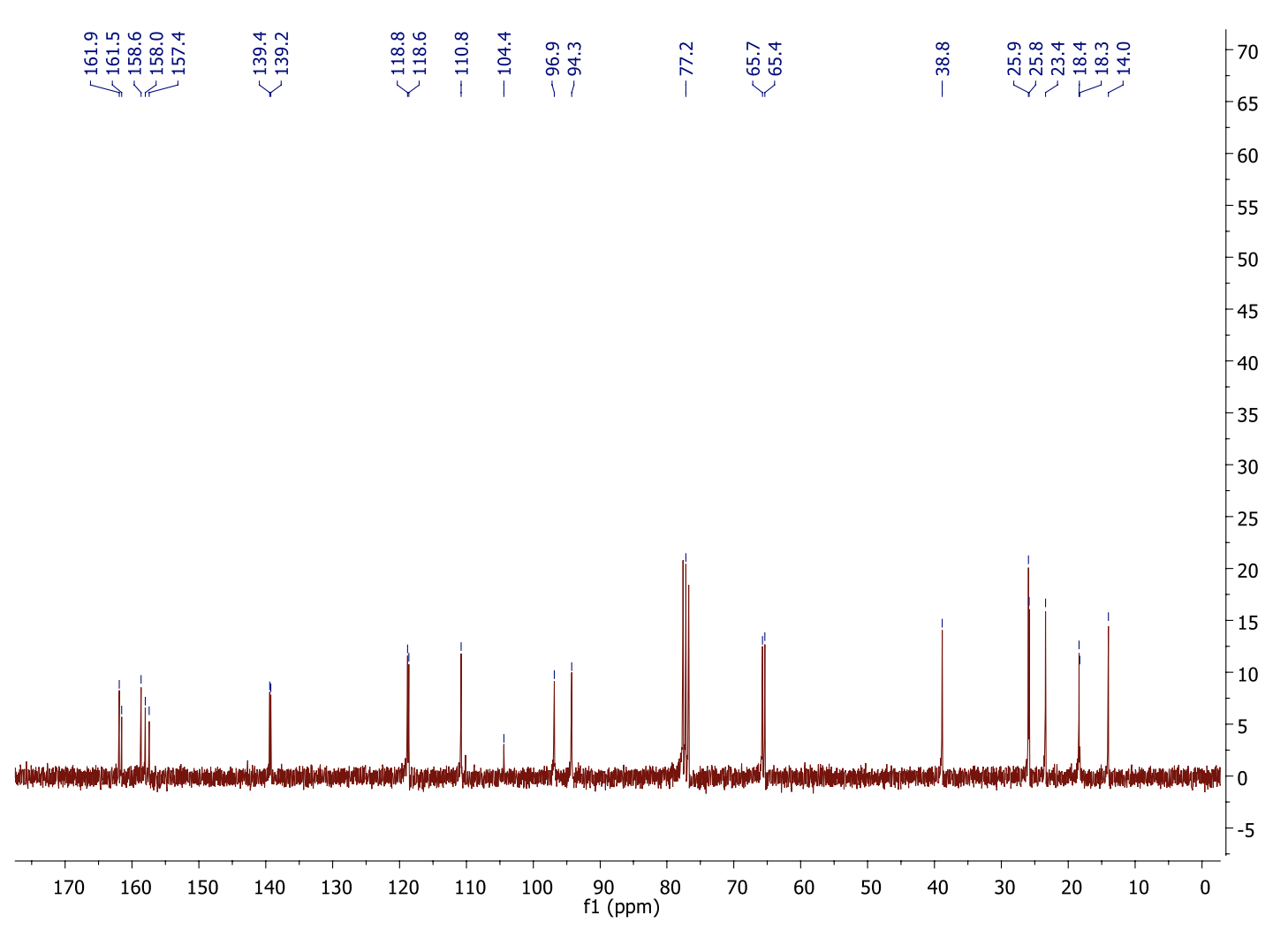


**5,7-digeranyloxy-4-propyl-2H-chromen-2-one (4i)**

**^1^H NMR**


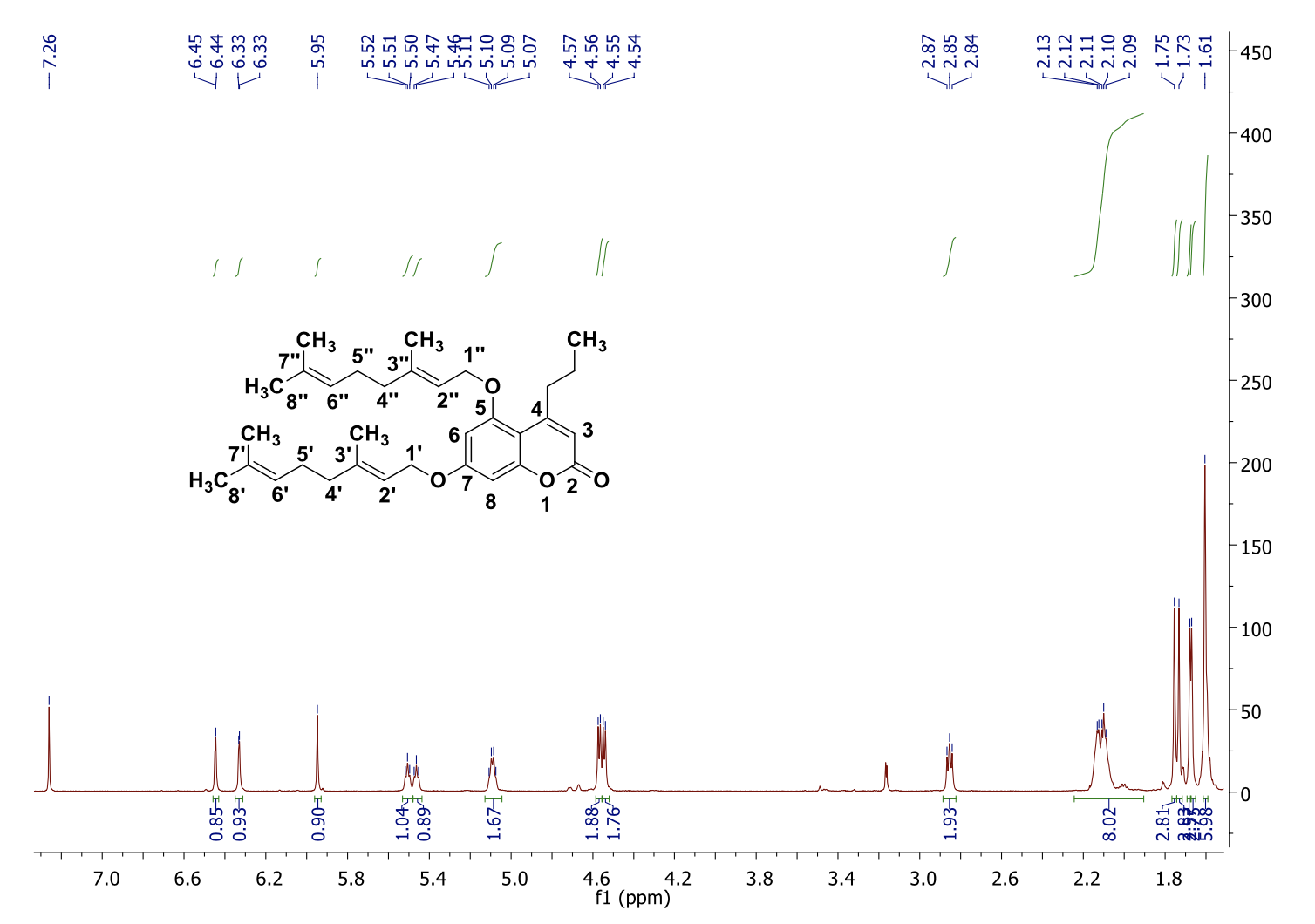


**^13^C NMR**


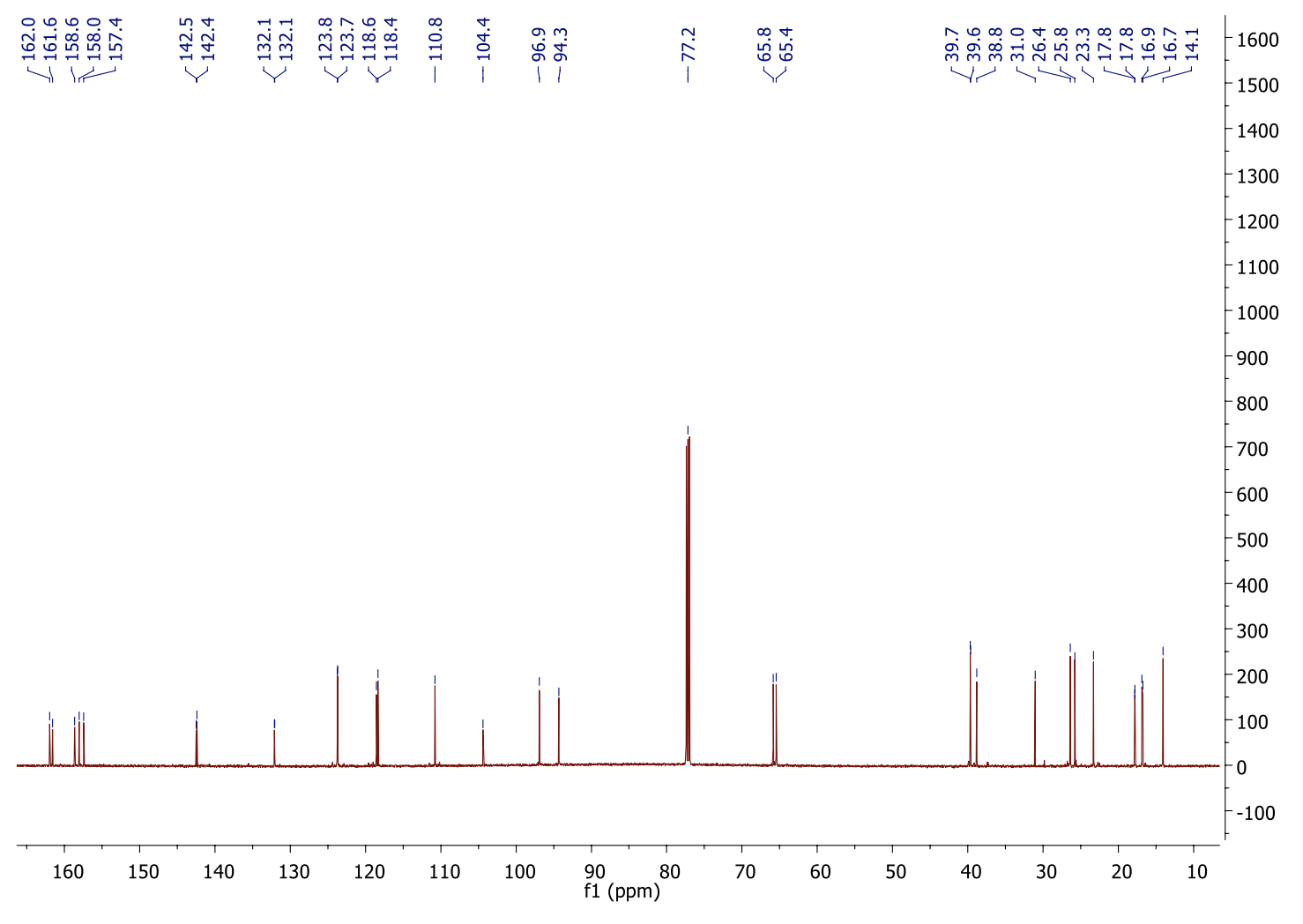


**7-prenyloxy-2H-chromen-2-one (4j)**

**^1^H NMR**


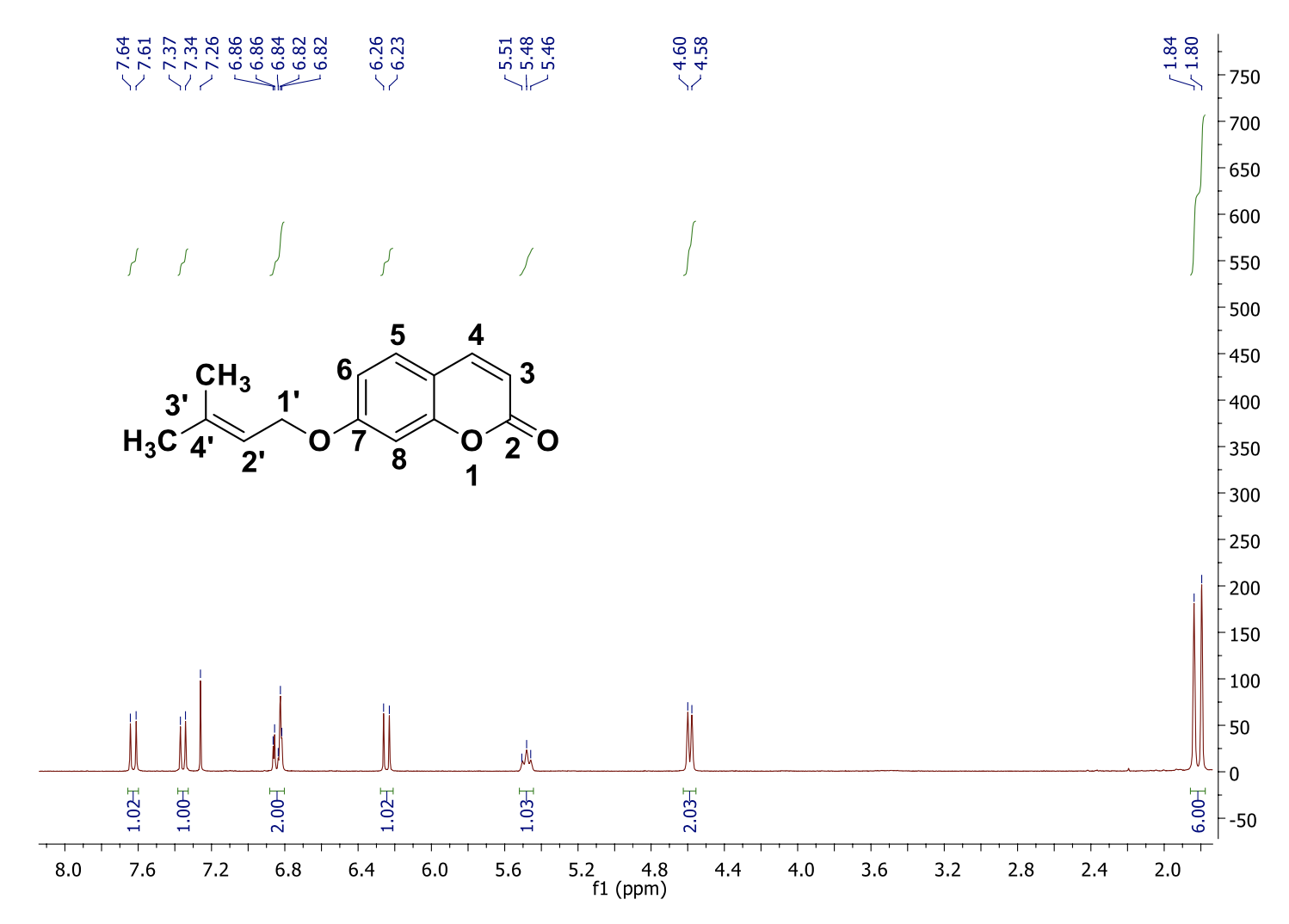


**7-geranyloxy-2H-chromen-2-one (Auraptene) (4k)**

**^1^H NMR**


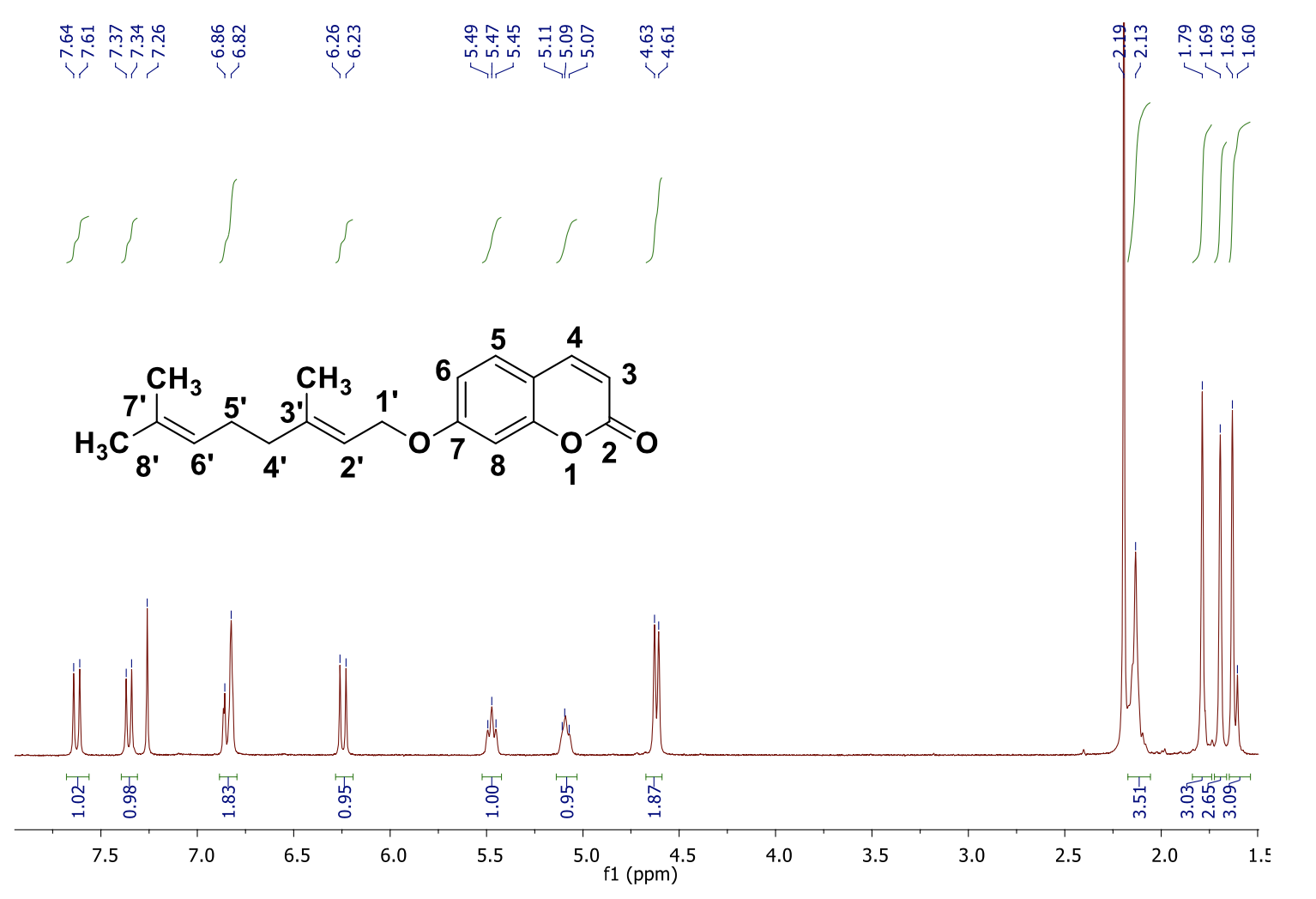


**7-farnesyloxy- coumarin (Umbelliprenin) (4l)**

**^1^H NMR**


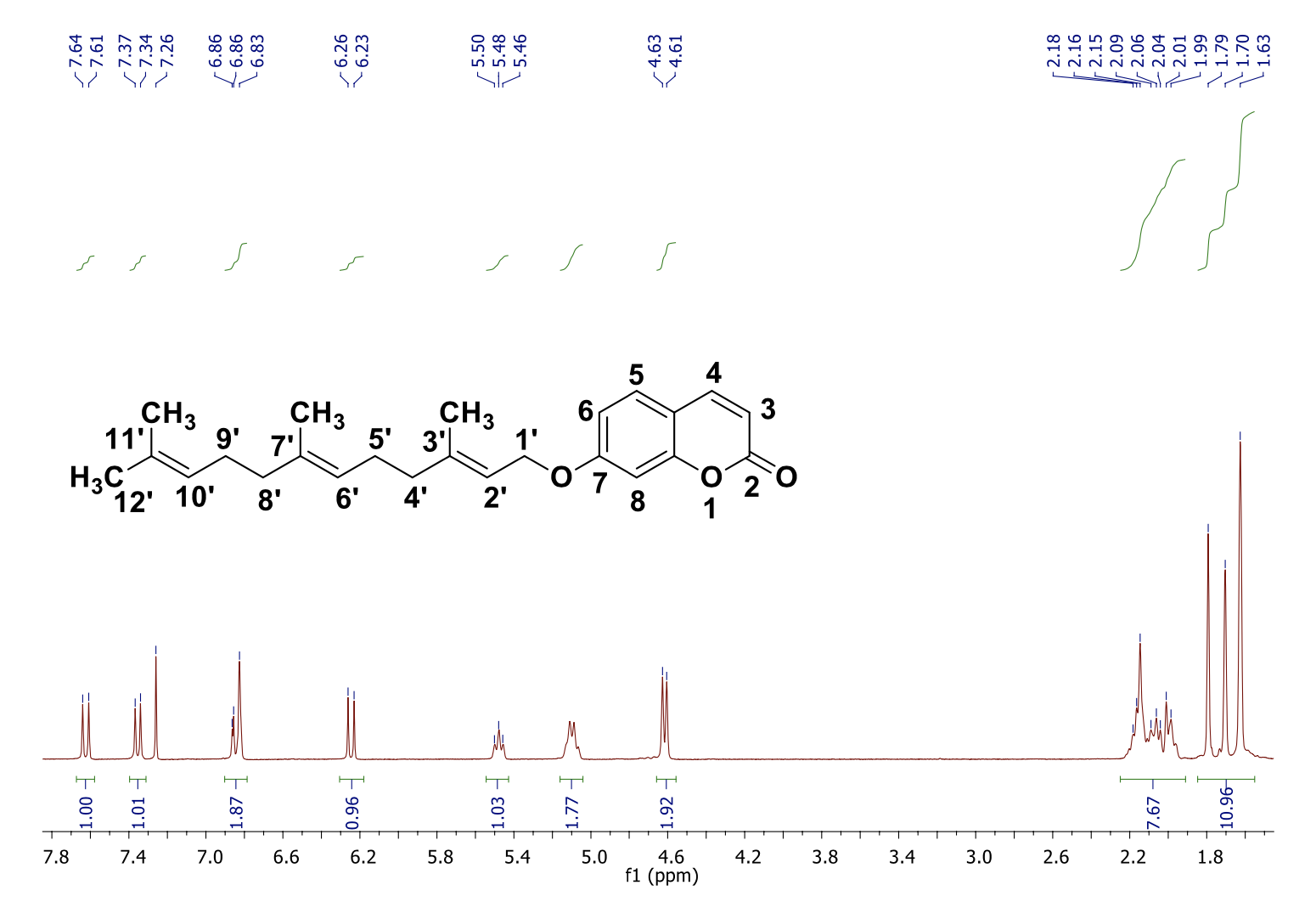


**7-geranylgeranyloxy-coumarin (4m)**

**^1^H NMR**


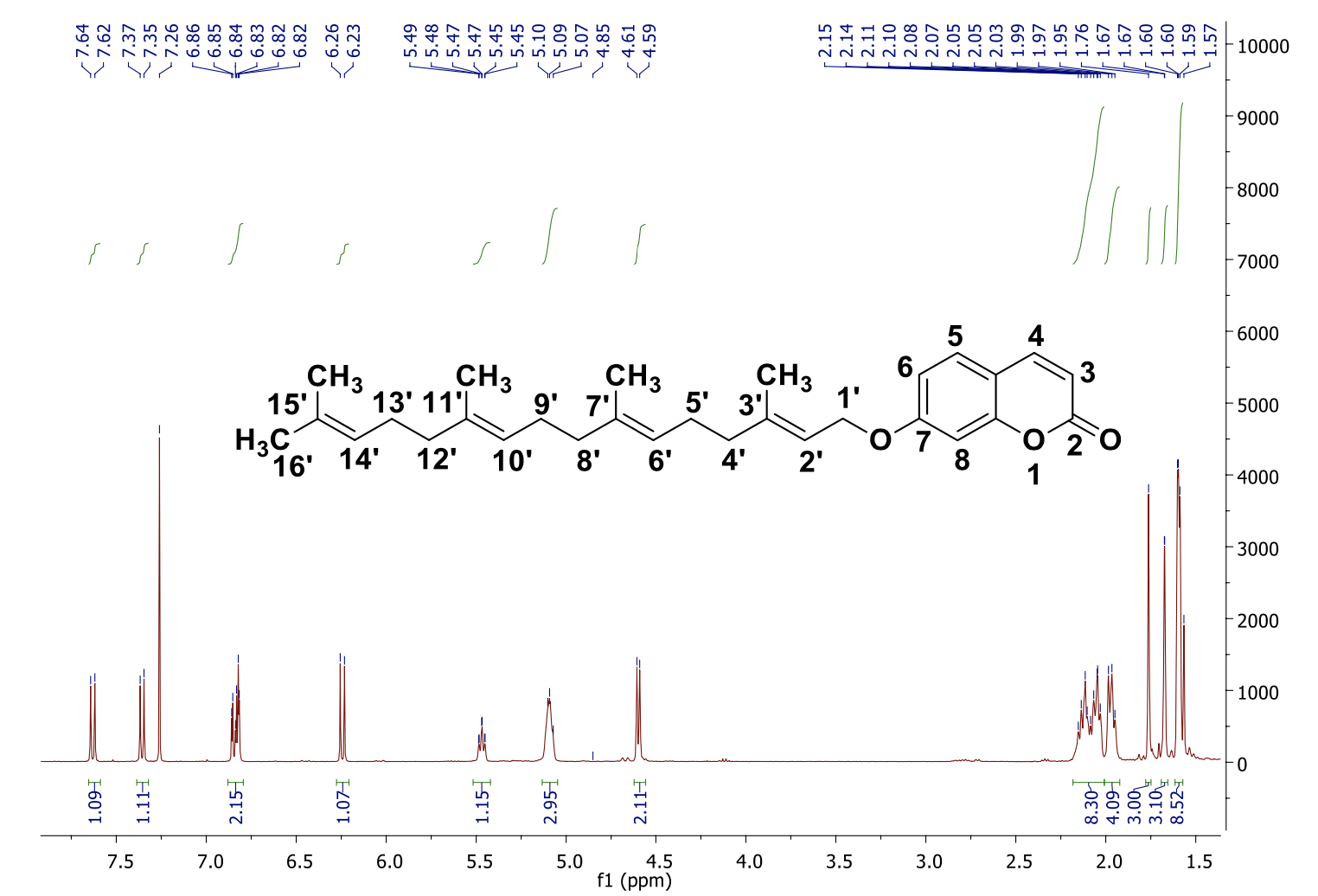


**5,7-diacetyloxy-4-methyl-2H-chromen-2-one (5)**

**^1^H NMR**


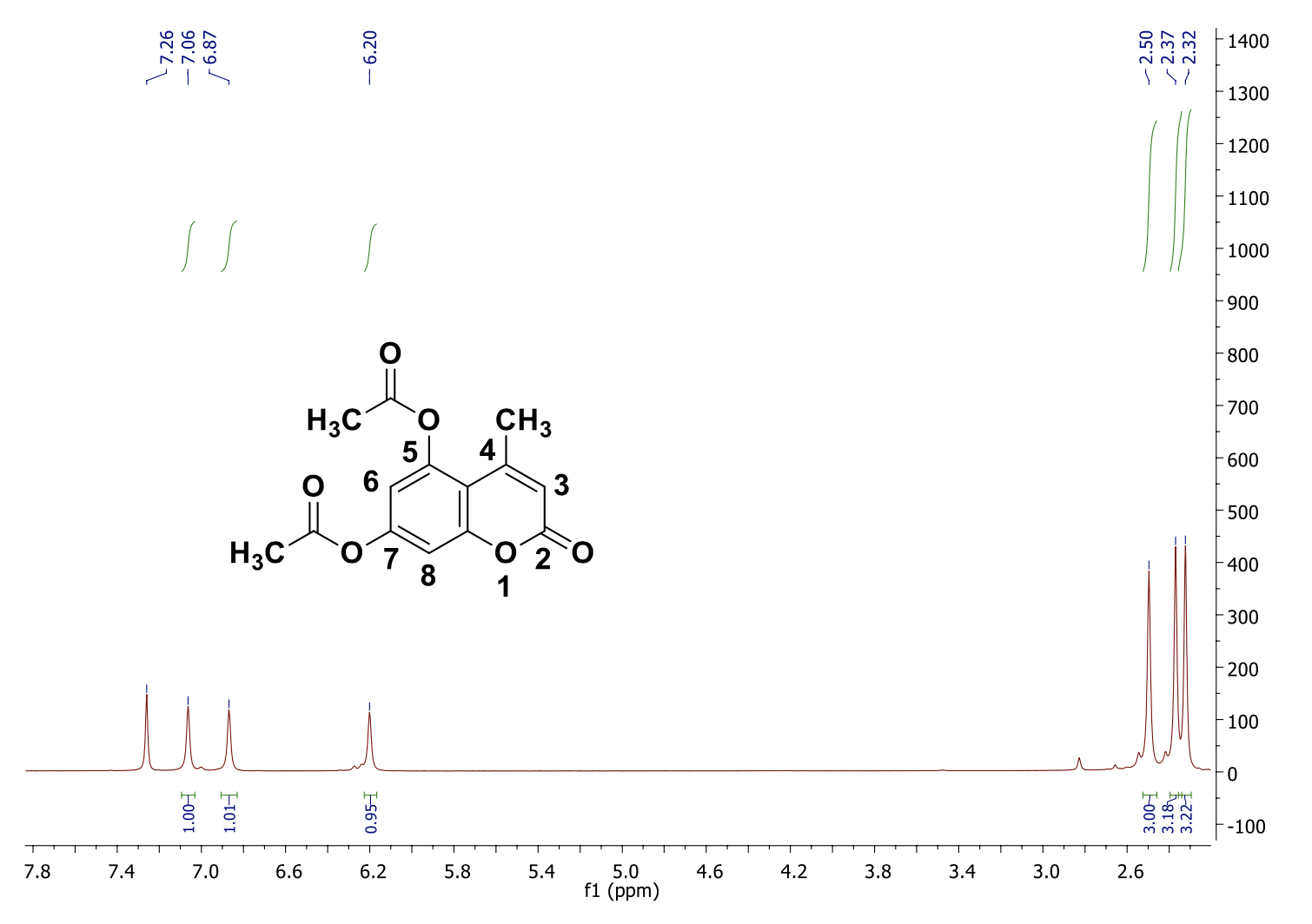

Supplement: Supplementary file 1 — Supplementary material 1 (DOCX 3770 kb) [file 11030_2020_10082_MOESM1_ESM.docx]
